# Increasingly severe thermal stresses on global photosynthesis using insights from observations of canopy temperature

**DOI:** 10.1093/nsr/nwag347

**Published:** 2026-06-22

**Authors:** Tianbo Pan, Hao Xu, Chris Huntingford, Shuchang Tang, Kai Wang, Josep Peñuelas, Shilong Piao

**Affiliations:** Sino-French Institute for Earth System Science, College of Urban and Environmental Sciences, Peking University, Beijing 100871, China; Sino-French Institute for Earth System Science, College of Urban and Environmental Sciences, Peking University, Beijing 100871, China; International Institute for Applied Systems Analysis, Laxenburg 2361, Austria; UK Centre for Ecology and Hydrology, Wallingford OX10 8BB, UK; Sino-French Institute for Earth System Science, College of Urban and Environmental Sciences, Peking University, Beijing 100871, China; Sino-French Institute for Earth System Science, College of Urban and Environmental Sciences, Peking University, Beijing 100871, China; CREAF, Cerdanyola del Vallès, Barcelona 08193, Spain; CSIC, Global Ecology Unit CREAF-CSIC-UAB, Barcelona 08193, Spain; Sino-French Institute for Earth System Science, College of Urban and Environmental Sciences, Peking University, Beijing 100871, China

**Keywords:** canopy temperature, gross primary productivity, optimum temperature, thermal acclimation, thermal stress

## Abstract

Temperatures could routinely exceed the optimal levels for photosynthesis as global warming intensifies, imposing thermal stress on the productivity of vegetation. We utilized satellite-derived canopy temperature and gross primary productivity data from 2003 to 2024 to identify the ecosystem-level optimal canopy temperature (${T}^{\rm can}_{\rm opt} $) for global photosynthesis and the extent of any thermal acclimation, which may offset warming impacts. Our findings indicate that across the globe, heat-induced restrictions on global photosynthesis are worsening, and areas subjected to thermal limitations have expanded by 1.7 billion hectares (57% increase) over the last 22 years. The number of days per year with high thermal suppression of photosynthesis during that period has increased sharply, averaging 28 days globally, and is especially high in tropical forests (117 days) and key agricultural regions (39 days). We demonstrate that vegetation acclimation to higher canopy temperature is partially mitigating emerging heat stress, but it is insufficient to keep up with the rate of global warming, with more than 90% of vegetated areas across the globe exhibiting only partial acclimation. A key feature of our analysis is the use of canopy-level temperatures, which more accurately represent the actual temperatures that vegetation physiologically responds to, rather than air temperature used in previous research. This difference accounts for our identified more rapidly intensifying vegetation response to warming than that estimated by other analyses. Overall, our canopy-level analysis reveals an escalating threat to global vegetation productivity and highlights the need for climate models to have refined land components, which often rely on air-temperature forcing and simplified acclimation schemes. Required are targeted ecosystem management strategies for adaptation to further global warming.

## Teaser

Satellite-derived canopy temperatures reveal a rapidly escalating heat threat to global photosynthesis over the past 22 years. Although vegetation can partly acclimate to rising temperatures, this adjustment is failing to keep pace with climate warming, leaving ecosystems increasingly vulnerable to heat-driven productivity losses.

## INTRODUCTION

Photosynthesis, as a fundamental component of plant growth and the carbon cycle, provides food and fibre that are essential for ecosystems and society [1,2]. The rate of photosynthetic carbon sequestration in plants is highly sensitive to global warming across scales, from individual leaves to entire ecosystems [3–5]. The temperature response of photosynthesis typically follows a unimodal curve that first increases relatively slowly with temperature until it exceeds the optimum (*T*_opt_) [6–8], after which the rate declines very rapidly. This substantial asymmetry implies that knowing *T*_opt_ is crucial, as should the temperature rise above it, then a strong suppression of photosynthesis will occur. Hence, insights into *T*_opt_ values provide vital information for assessing the margin of safety between current warming levels and the higher optimal temperature [9–12], or the extent to which thermal limits have been reached [13,14] under climate change. Heatwaves have become more frequent and widespread in recent decades, and in tandem with ongoing background climatic warming, they have caused many instances of observed severe suppression of vegetation productivity and even large-scale die-offs [15–17]. These rapidly escalating heatwaves and associated extreme temperature events underscore, in particular, the pressing need for a thorough data-led evaluation of the current state and changes in thermal limitation risk for global vegetation.

Field experiments and biochemical models suggest that plants can partially adapt to varying background temperature levels by self-regulation [6,18]. For example, tropical plants exhibit a higher *T*_opt_ than plants in temperate and boreal regions [8,19]. *T*_opt_ can also increase (i.e. acclimate) with rising temperature and the concentration of atmospheric carbon dioxide (CO_2_), alleviating any potential negative impacts of human-induced global warming on photosynthesis [12,20]. However, considerable debate persists regarding whether global vegetation can fully acclimate to changes in temperature on a decadal scale, and if not, then what is the extent of partial acclimation that may occur [8,21,22]. Previous studies have primarily relied on space-for-time substitution to estimate rates of acclimation [8,12], which implicitly assume that vegetation thermal optima are in (or close to) equilibrium with the local climate (i.e. near-unity tracking of warming). However, spatial variation in *T*_opt_ largely reflects long-term adaptation to the climate of origin, whereas temporal changes over recent decades are constrained by shorter-term physiological acclimation and ecological limits [23,24]. As a result, space-for-time approaches may overestimate the capacity of *T*_opt_ to track rapid contemporary warming. Furthermore, while leaf- and site-level studies provide critical physiological insights [25–28], ecosystem-scale assessments remain scarce. Few studies explicitly assess whether observed temporal adjustments in *T*_opt_ actually reduce exposure to supra-optimal heat stress. Taken together, these gaps reflect an insufficient understanding of the short-term thermal acclimation of ecosystem-scale *T*_opt_, which in turn greatly impedes our ability to accurately quantify the capacity of vegetation to cope with a warming world.

Much of our knowledge of ecosystem-level *T*_opt_ arises from studies examining the response of photosynthesis to ambient air temperature (*T*_air_) [8,12,21,22]. Although this approach yields valuable information, photosynthesis is more directly governed by the leaf temperature (*T*_leaf_) [29] or canopy temperature (*T*_can_). Substantial evidence shows that *T*_can_ can significantly differ from air temperature due to the influence of environmental controls (e.g. water stress and solar radiation), foliar traits, and canopy structure [28–33]. While transpirational cooling and microclimatic buffering can maintain *T*_can_ below or increase more slowly than *T*_air_ [34,35], intense solar radiation can conversely drive *T*_can_ above or warm faster than ambient air temperatures [36,37]. These driving mechanisms often coexist, exhibiting complex spatial and temporal heterogeneity in the patterns of canopy temperature [36,38]. With the continuously expanding availability of canopy temperature observations from thermal cameras and satellites [13,31], we now have the opportunity to gain novel insights into the more physiologically relevant response of photosynthesis to temperature at the canopy scale.

Here, we used simultaneous satellite-derived observations of canopy temperature (*T*_can_) and gross primary productivity (GPP) to investigate how photosynthesis has responded to changes in canopy thermal conditions over the last two decades (2003–24) (see Methods). Although the temperature response of photosynthesis is ultimately determined at the leaf scale, we emphasize that the *T*_can_ used here represents an ecosystem-scale, spatially integrated radiometric signal of the vegetation canopy rather than the temperature of individual leaves or specific canopy layers. It serves as a robust proxy for the thermal environment experienced by vegetation and covaries closely with leaf temperature over broad spatio-temporal scales. Consistent with this, both canopy temperature and vegetation productivity in our study represent spatially and vertically integrated canopy- to ecosystem-scale signals rather than leaf-scale measurements.

Using the full record, we first characterized the global distribution of the optimal canopy temperature for ecosystem photosynthesis (${T}^{\rm can}_{\rm opt} $) and explored its dependence on background climatic conditions. We then quantified local shifts in ${T}^{\rm can}_{\rm opt} $ over the last 22 years to assess whether thermal acclimation has occurred. Finally, we evaluated changes in the magnitude and range of thermal conditions experienced by global vegetation, with a particular focus on the extent and frequency of supra-optimal heat stress and on whether acclimation has kept pace sufficiently to constrain such stress. To contextualize our findings, we compared our results against traditional *T*_air_-based assessments in our analysis to investigate how reliance on *T*_air_ may have influenced previous estimates [8,12,21,22] relative to the actual thermal conditions experienced by plant canopies. The robustness of our results was further verified using three alternative GPP proxies: the near-infrared reflectance of vegetation (NIR_V_) [39], its photosynthetically active radiance-multiplied product (NIR_V_P) [40], and the contiguous solar-induced chlorophyll fluorescence (CSIF) [41]. In addition, we independently evaluated the satellite-derived *T*_can_ against site-scale aerodynamic canopy temperature estimates derived from eddy-covariance observations. By integrating a physiologically grounded canopy-temperature framework, high-resolution (1-km) observations, and extended temporal coverage, our study aims to advance previous researches [8,12,21,22], providing an explicit assessment of how vegetation thermal exposure and acclimation capacity are evolving in a warming world.

## RESULTS

### Spatial distribution of ${T}^{\rm can}_{\rm opt} $

**Figure 1.**
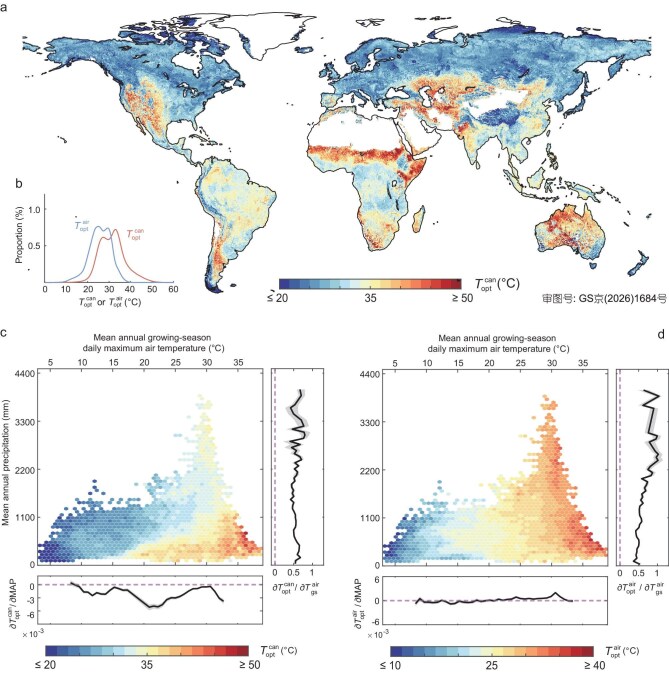
Distributions of derived ${T}^{\rm can}_{\rm opt} $ and ${T}^{\rm air}_{\rm opt} $ values for vegetation productivity and their connection to background climate. (a) Spatial distribution of ${T}^{\rm can}_{\rm opt} $ for photosynthesis determined using simultaneous canopy temperature and GPP values based on satellite observations from the Moderate Resolution Imaging Spectroradiometer (MODIS). The satellite data used covers the years 2003–24 inclusive. Values are only presented for vegetated areas [i.e. for annual mean normalized difference vegetation index (NDVI) values larger than 0.1], where a value of ${T}^{\rm can}_{\rm opt} $ is detected and where the growing season lasts longer than one month; otherwise, the location is blank (i.e. white). (b) The probability-density functions (expressed as percentages) represent the distributions of ${T}^{\rm can}_{\rm opt} $ and $T^{\rm air}_{\rm opt}$ (shown in panel a and Fig. S1a, respectively), based on the proportions of their respective actual areas relative to the total vegetated area. The blue line represents the same distribution but for ${T}^{\rm air}_{\rm opt} $, with the detailed spatial pattern shown in Fig. S1a. (c) The climatic dependence of ${T}^{\rm can}_{\rm opt} $ on air temperature and precipitation, along with relative sensitivities (side panels). Note the ‘*x*’-axis label and values are presented across the top of the main panels (c and d). Each climatic bin was defined by intervals of 0.7°C of mean annual growing-season daily maximum air temperature (${T}^{\rm air}_{\rm gs} $) and 70-mm intervals of mean annual precipitation (MAP), based on current climatic conditions averaged between 2003 and 2024. The solid line in the extra bottom (extra right) panel represents the sensitivity to temperature (precipitation) along the precipitation (temperature) gradient, calculated as the slope of the linear regression between ${T}^{\rm can}_{\rm opt} $ and ${T}^{\rm air}_{\rm gs} $ (MAP) for a given level of precipitation (temperature). The shaded area indicates the standard deviation of the sensitivity (∂${T}^{\rm can}_{\rm opt} $/∂${T}^{\rm air}_{\rm gs} $ or ∂${T}^{\rm can}_{\rm opt} $/∂MAP) estimated by bootstrapping. (d) Similar to (c), but instead makes all calculations of *T*_opt_ based on air temperature rather than canopy temperature, and therefore presents variations in ${T}^{\rm air}_{\rm opt} $.

We identified the global pattern of ${T}^{\rm can}_{\rm opt} $ for photosynthesis by using simultaneous satellite observations of canopy temperature and GPP data from 2003 to 2024 inclusive (see Methods). The average ${T}^{\rm can}_{\rm opt} $ across all global vegetated areas was estimated to be 30 ± 6°C (mean ± 1 standard deviation), with 98% of the areas falling within the range of 20–50°C (Fig. 1a and b). ${T}^{\rm can}_{\rm opt} $ exhibited substantial geographical heterogeneity in its spatial distribution, as shown in Fig. 1a. Along latitudinal gradients, higher values of ${T}^{\rm can}_{\rm opt} $ were found in tropical regions (33 ± 4°C) compared to polar regions (25 ± 4°C), which are generally warmer locations due to more intense solar radiation. Arid regions such as the Sahel, the Somali Peninsula, the central United States of America, central Asia, and northern Australia had especially high ${T}^{\rm can}_{\rm opt} $ values nearing 50°C, likely due to the additional effect of limited water availability. In addition, lower values of ${T}^{\rm can}_{\rm opt} $ close to 20°C were observed in high-altitude regions like the Tibetan Plateau and the Andes in Chile. We also estimated the global optimal air temperature (${T}^{\rm air}_{\rm opt} $) for photosynthesis using air temperature from the ECMWF Reanalysis v5 (ERA5-Land) product (Fig. S1a). ${T}^{\rm can}_{\rm opt} $ and ${T}^{\rm air}_{\rm opt} $ exhibited similar spatial patterns, but ${T}^{\rm air}_{\rm opt} $ (25 ± 5°C) was substantially lower than ${T}^{\rm can}_{\rm opt} $ (Figs. 1b and S1a). This discrepancy occurred because the cooling effect of foliar transpiration during daytime photosynthesis, particularly under strong midday sunlight, is insufficient to counteract radiative heating [35,36], thereby leading to canopy temperatures that exceed the ambient air temperature. This difference was especially pronounced in arid regions (Fig. S1b), where it could exceed 15°C due to further restrictions on transpiration.

We then analysed how optimal temperatures depend on background precipitation levels and temperature forcings. Hence, in Fig. 1c and d, we present ${T}^{\rm can}_{\rm opt} $ and ${T}^{\rm air}_{\rm opt} $ as functions of the climatic space defined by both mean growing-season daily maximum air temperature (${T}^{\rm air}_{\rm gs} $) and mean annual precipitation (MAP). Both ${T}^{\rm can}_{\rm opt} $ and ${T}^{\rm air}_{\rm opt} $ increased with background temperature around the globe, consistent with both previous studies attributing this relationship to evolutionary adaptation [6–8,42] and features of Fig. 1a. The spatial sensitivities of ${T}^{\rm can}_{\rm opt} $ and ${T}^{\rm air}_{\rm opt} $ to ${T}^{\rm air}_{\rm gs} $ were positive but less than unity for most precipitation bins (right-hand panels of Fig. 1c and d), suggesting that spatial gradients of ${T}^{\rm can}_{\rm opt} $ generally follow, but do not fully keep pace with the increases in background temperature. This difference may reflect hydraulic and phenological limitations, or lower interannual variability in warmer region [43]. The sensitivities of ${T}^{\rm can}_{\rm opt} $ and ${T}^{\rm air}_{\rm opt} $ to background temperature were robust (Fig. S2), regardless of how background temperature was defined, including whether daily maximum rather than daily average temperature was used, or whether annual rather than growing-season data were used.

Notable, ${T}^{\rm can}_{\rm opt} $ consistently exhibited negative (i.e. decreasing) sensitivity to increasing background precipitation across most temperature ranges (Fig. 1c). This suggests that vegetation in drier environments can tolerate high foliar temperature extremes by having higher ${T}^{\rm can}_{\rm opt} $ values (as also reflected geographically in higher ${T}^{\rm can}_{\rm opt} $ values; Fig. 1a). Plants in arid regions often close their stomata, or reduce stomatal density, to minimize the loss of water, but this also results in reduced transpirational cooling and consequently higher foliar surface temperatures [10,44,45]. Given this trade-off, plants tend to adapt by allowing higher foliar temperatures rather than potentially dying from the loss of water, and this may also explain the higher ${T}^{\rm can}_{\rm opt} $ values in such locations. Our findings in Fig. 1a and c suggest the capability of plants to enhance their heat tolerance by physiological and morphological adjustments, or by altering community composition, such as by increasing the proportion of heat-tolerant C_4_ plants [46]. Canopy temperature therefore effectively integrates the effects of both ambient air temperature and water stress, to give a reliable estimate of optimal temperature, *T*_opt_, as a function of both these climatological drivers. This negative sensitivity of ${T}^{\rm can}_{\rm opt} $ to MAP remains consistent across different background temperatures (Figs S2 and S3). However, ${T}^{\rm air}_{\rm opt} $ depended only weakly on spatial variations in precipitation, with sensitivities close to or slightly above zero across most temperature bins (Fig. 1d). This finding suggests that air temperature does not capture the adaptation of vegetation to water stress, likely due to its poor representation of the physiological and structural responses of vegetation to environmental change [31,38]. These errors matter, as in a changing climate that is altering both temperature and aridity levels, it is important to avoid compensating errors appearing in any dependencies of calibrated parameters of vegetation models, such as *T*_opt_. These findings were also consistently reproduced when using alternative GPP proxies (NIR_V_, NIR_V_P and CSIF; Figs S4–S6).

### Thermal acclimation of ${T}^{\rm can}_{\rm opt} $

**Figure 2.**
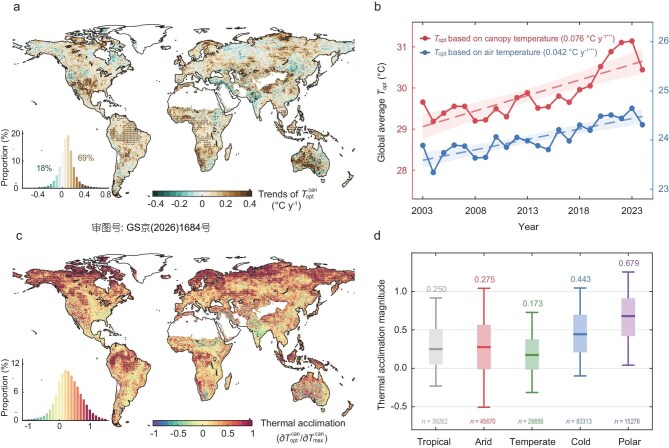
Temporal changes of ${T}^{\rm can}_{\rm opt} $ and its magnitude of thermal acclimation over the last two decades. (a) Spatial pattern of trends in ${T}^{\rm can}_{\rm opt} $ from 2003 to 2024. Dot markers (·) denote grid regions where the trend is significant at the 0.05 level (*P* < 0.05). The inset histogram shows the areal proportion across different trend magnitudes, with the total percentage annotated. (b) The temporal changes of globally averaged ${T}^{\rm can}_{\rm opt} $ and ${T}^{\rm air}_{\rm opt} $ for the period 2003–24. The solid line shows annual global means, and the dashed line represents the trend fitted using least squares regression and its 95% confidence interval as a grey plume. ***P < 0.001. (c) Spatial distribution of the magnitude of thermal acclimation, defined as the ratio ∂${T}^{\rm can}_{\rm opt} $/∂${T}^{\rm can}_{\rm max} $ over the last 22 years at each grid point. Regions with significant ratios (*P* < 0.05) are marked with dot symbols. The inset histogram shows the distribution of acclimation magnitudes. (d) Difference in thermal acclimation magnitude across major climate zones. Horizontal lines denote medians, boxes represent interquartile ranges, and whiskers span the 5th–95th percentiles. Median values are displayed above each box, and sample sizes (*n*) are shown below. Climate zones were defined according to the 1991–2020 Köppen–Geiger climate classification [51].

The optimal temperature for global photosynthesis is not static, and this holds particular importance in the context of rapid climate warming caused by human burning of fossil fuels. We found that the ${T}^{\rm can}_{\rm opt} $ value for vegetation productivity increased significantly from 2003 to 2024 at both regional and global scales. The globally averaged value of ${T}^{\rm can}_{\rm opt} $ increased by ∼0.076°C y^−1^ over the last 22 years (Fig. 2b). Regionally, ${T}^{\rm can}_{\rm opt} $ tended to increase with statistical significance (i.e. with *P* < 0.05 for the fitted trend) in 19% of vegetated areas worldwide, while it decreased significantly in only about 0.01% of the vegetated areas (Fig. 2a). Optimal temperatures exhibited a more widespread significant increase in tropical regions (0.083°C y^−1^) and so at a rate higher than the global average. In some high-latitude areas and arid regions, the rate of increase was even more rapid, reaching up to 0.4°C y^−1^, though substantial spatial heterogeneity was observed in both instances. In comparison, the global increase in estimated *T*_opt_ when derived from air temperature was about 0.042°C y^−1^, suggesting, possibly incorrectly, markedly slower changes in estimated optimal canopy temperature (Figs 2a and S7).

A key question is whether the observed changes in *T*_opt_ have tracked global warming and hence demonstrated a capacity to acclimate thermally to anthropogenic climate change. To assess this, we quantified the magnitude of thermal acclimation for each vegetation grid cell as the ratio ∂${T}^{\rm can}_{\rm opt} $/∂${T}^{\rm can}_{\rm max} $, derived from the regression coefficient between ${T}^{\rm can}_{\rm opt} $ and ${T}^{\rm can}_{\rm max} $ over the 22-year study period (Fig. 2c). Globally, we found that thermal acclimation is overwhelmingly partial. Vegetation exhibited an average acclimation magnitude of 0.37°C per 1°C, with over 90% of vegetated areas falling below unity (the 1:1 tracking benchmark). This empirical evidence at the ecosystem scale confirms that vegetation has only partially kept pace with rising heat stress, a finding remarkably consistent with established leaf-level observations [24–27]. Spatially, thermal acclimation was strongest and most significant in high-latitude regions (Fig. 2c and d), likely driven by large interannual temperature variability [22], warming-induced stimulation of nitrogen mineralization [48], and rapid shifts in species composition toward more productive functional groups [49]. This suggests that vegetation in these regions may be better able to cope with intensifying heat stress. Tropical rainforests also exhibited spatially widespread significant acclimation (Fig. 2c); however, it failed to fully track the imposed climate change. Because ambient temperatures in these biomes are already approaching critical physiological thresholds [13], such partial adjustment provides only limited protection against future warming. In contrast, most other regions showed weak or insignificant acclimation. Such limited responses could arise from insufficient temporal changes in canopy temperature to trigger acclimation [8] or from inherent physiological and ecological constraints that prevent thermal adjustment on decadal timescales. Moreover, species with broad photosynthetic thermal response curves may not need to shift *T*_opt_ [50], as moderate temperature changes do not substantially suppress their photosynthetic capacity. Finally, thermal acclimation estimated from air temperature exhibited similar spatial patterns, confirming the overall incomplete acclimation of vegetation to warming (Fig. S8). However, magnitudes based on air temperature were generally lower in tropical, arid, and temperate regions, but higher at high latitudes, reflecting regional biases when using air temperature to assess acclimation. These findings remained highly consistent across multiple GPP proxies (NIR_V_, NIR_V_P, and CSIF; Figs S9–S11), confirming the robustness of our finding that global vegetation acclimation is insufficient to keep pace with a warming climate.

### Expansion of thermally limited areas

**Figure 3.**
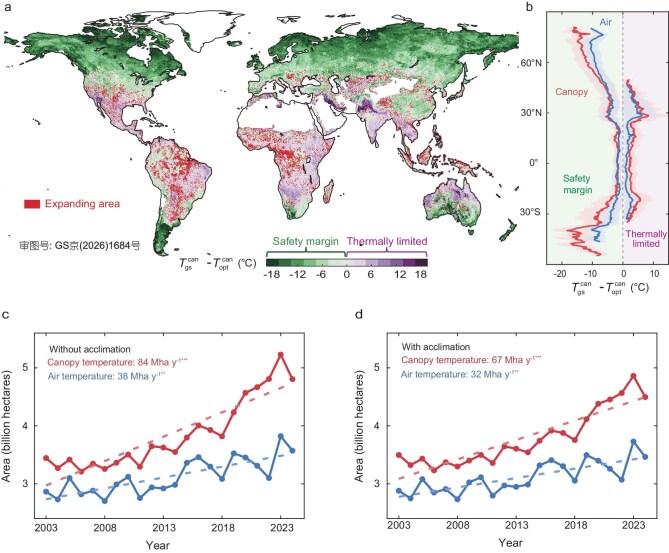
Patterns of the difference between ${T}^{\rm can}_{\rm opt} $ and ${T}^{\rm can}_{\rm gs} $, and the expansion of thermally limited areas at the canopy scale. (a) The difference between the optimal canopy temperature (${T}^{\rm can}_{\rm opt} $) (and so calculated similarly to the ${T}^{\rm can}_{\rm opt} $ values in Fig. 1, and not including acclimation) and the multiyear mean growing-season average daily maximum canopy temperature (${T}^{\rm can}_{\rm gs} $) during the period 2003–24 inclusive. When ${T}^{\rm can}_{\rm opt} $ is higher than ${T}^{\rm can}_{\rm gs} $, the difference represents a safety margin for vegetation productivity under warming; conversely, when ${T}^{\rm can}_{\rm gs} $ exceeds ${T}^{\rm can}_{\rm opt} $, the area is already subject to thermal stress, with the difference illustrating the extent of thermal limitations on photosynthesis. This stress is due to the asymmetry of the photosynthetic response, where even a small increment in temperature above ${T}^{\rm can}_{\rm opt} $ results in a disproportionately large suppression of productivity. The expansion of thermally limited areas without acclimation is depicted in panel (a) as an overlay, calculated as areas where $T^{\rm can}_{\rm gs}$ was lower than $T^{\rm can}_{\rm opt}$ during the first five years (2003–7) but exceeded $T^{\rm can}_{\rm opt}$ in the last five years (2020–24), corresponding to the line based on canopy temperature in panel (c). (b) The latitudinal average of ${T}^{\rm can}_{\rm opt} $- ${T}^{\rm can}_{\rm gs} $ based on canopy and air temperatures. Regions with safety margins and thermal limitations are calculated separately to prevent the cancellation of positive and negative values. The combined distributional pattern of ${T}^{\rm air}_{\rm opt} $-${T}^{\rm air}_{\rm gs} $ is shown in Fig. S12. (c and d) Yearly changes over the last 22 years in the total area of thermally limited regions where the mean growing-season temperature exceeded its optimal value (i.e. where ${T}^{\rm can}_{\rm gs} $ > ${T}^{\rm can}_{\rm opt} $). Panel (c) illustrates changes without considering thermal acclimation, and panel (d) incorporates acclimation. In panels (c and d), the dashed lines represent linear-trend fits. **P < 0.01; ***P < 0.001.

To move beyond mapping ${T}^{\rm can}_{\rm opt} $ patterns and trends like in previous studies [8,21], we explicitly quantified supra-optimal heat stress exposure across global vegetation. We analysed the difference between ${T}^{\rm can}_{\rm opt} $ and ${T}^{\rm can}_{\rm gs} $ from the years 2003 to 2024 and across global vegetation, to determine whether high temperatures have become a limiting factor for canopy photosynthesis. Given the strong nonlinearity at high temperatures in the photosynthetic response, we are particularly interested in whether ${T}^{\rm can}_{\rm gs} $ > ${T}^{\rm can}_{\rm opt} $. Our findings illustrate that the multi-year mean ${T}^{\rm can}_{\rm gs} $ under current mean-decadal climatic conditions is substantially higher than the value of ${T}^{\rm can}_{\rm opt} $ in arid and semi-arid regions such as Central America, the southeastern Amazon Basin, African savannas, the Somali Peninsula, India, Australia, and central Asia (Fig. 3a and b). This suggests that photosynthesis in these areas has been persistently constrained by thermal stresses (Fig. 3a and b). And the optimal temperature in the three major tropical rainforest regions was already slightly lower (1.0 ± 2.0°C) than the average temperature during the growing season (Fig. 3a), leaving no remaining safety margin for photosynthesis and posing an even greater risk especially if any acclimation does not keep pace with future warming. In contrast, mid- to high-latitude regions in both hemispheres still retained a large safety margin, where warming could continue to enhance photosynthesis (Fig. 3a and b). The pattern of differences between optimal and average air temperature was similar to that when using canopy temperatures in calculations (Fig. S12), although the level of thermal limitation was less pronounced (Fig. 3b).

The areas subjected to normalized thermal limitation have gradually expanded over the last 22 years as warming has intensified (red overlay in Fig. 3a, with the definition in the caption; see also Fig. 3c). Globally, areas where the average growing-season canopy temperature (${T}^{\rm can}_{\rm gs} $) exceeded ${T}^{\rm can}_{\rm opt} $ have increased from ∼3.0 billion to 4.7 billion hectares during that period if acclimation were not considered (and thus an annual rate of increase of 84 hectares Mha y^−1^; Fig. 3c, red curve). This expansion has notably accelerated in the last decade (Fig. 3c). As might be expected, most of the expansion occurred at the edges of existing thermally limited areas, with widespread expansion in the southern Amazon Basin, the African Sahel and southern Africa, Madagascar, and Mexico. Additionally, despite the significant capability of ${T}^{\rm can}_{\rm opt} $ to acclimate to recent background warming in many locations (Fig. 2), this physiological adjustment has only marginally decelerated the expansion of these thermally limited areas over the last two decades. Specifically, acclimation has slowed the rate of expansion from 84 to 67 Mha y^−1^ by adjusting ${T}^{\rm can}_{\rm opt} $. The rate of expansion would still exceed estimates based on air temperature (38 Mha y^−1^ without acclimation and 32 Mha y^−1^ with acclimation). Comparable results were obtained when using alternative GPP proxies (Figs S13–S15).

### The increasing frequency of thermal limitations

**Figure 4.**
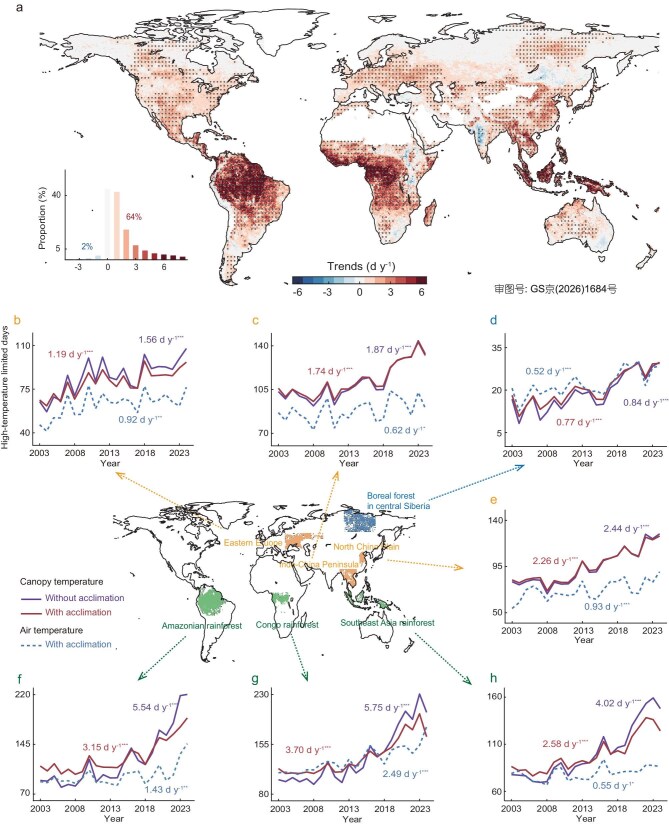
Trends in the number of high-temperature days over the past 22 years, with potential to suppress vegetation productivity. (a) Spatial distribution of trends in the frequency of days on which photosynthesis is subjected to thermal limitation during the growing period and from years 2003 to 2024. Calculations are based on crossing derived spatially specific values of ${T}^{\rm can}_{\rm opt} $, but assuming the latter are invariant in time, and thus without thermal acclimation. Trends were calculated using linear regression and the standard least squares method, and regions with significant trends (*P* < 0.05) were marked with dot symbols. The inset histogram represents the areal proportion for different trend magnitudes, binned in intervals of 1 d y^−1^. (b–h) Interannual variations and trends in the number of high-temperature limited days for seven key regions as marked on the map: three agricultural regions, including eastern Europe, Indo-China Peninsula and the North China Plain, boreal forests in central Siberia, three tropical rainforest regions including the Amazon basin, Congo and Southeast Asia. In each panel, curves are shown for calculations based on canopy temperature without acclimation in $T^{\rm can}_{\rm opt}$, canopy temperature with acclimation in $T^{\rm can}_{\rm opt}$ and air temperature with acclimation in $T^{\rm air}_{\rm opt}$. Statistical significance levels for all text annotations are: *P < 0.1; **P < 0.01; and ***P < 0.001.

In addition to our derivation above of the expansion of thermally limited areas, we also considered temporal variations for particular fixed locations. Specifically, we analysed the changes in the number of days within the growing season each year when canopy temperature exceeded *T*_opt_ levels, thereby impacting vegetation growth and photosynthesis. It is noted that there may not be a simple one-to-one correspondence between rates of background warming and increases in extreme events, caused by a straightforward shift in the distribution of daily temperatures. Instead, for some locations, very high-temperature events have recently accelerated relative to general warming changes [52]. Although the number of extremely high-temperature days in our study was low in regions such as tropical rainforests and high-latitude regions around the globe (Fig. S16), high-temperature events are increasing in their occurrence under recent warming. The global average number of days when heat could potentially suppress productivity for vegetation has increased at a rate of 1.26 d y^−1^ from 2003 to 2024 although this statistic is derived without considering thermal acclimation (Fig. 4a). During this period, 64% of global vegetated areas experienced a statistically significant increase in the number of high-temperature events, with rates from 0.38 to 6.92 d y^−1^, corresponding to the 95% confidence interval (dot markers and positive values; Fig. 4a).

Tropical forests, in particular, have experienced a substantial and widespread increase in the number of high-temperature limited days (Fig. 4f–h), especially when not accounting for acclimation. This finding suggests that global warming has the potential to cause much more routine overriding of safe thermal margins in those locations. In the Amazon rainforest (Fig. 4f), high-temperature limited days have increased by nearly 120 days over the last 22 years. In earlier periods, these high-temperature episodes often coincided with major El Niño events, such as in 2005, 2010, and 2015/2016. The rate of increase in canopy temperature over the last 5 years, however, has far outpaced the rate in previous years, and this cannot be linked to natural variation via El Niño alone. The frequency of possibly damaging high-temperature events also increased substantially in the Congo rainforest (Fig. 4g) and forests in southeastern Asia (Fig. 4h), particularly over the last decade. The thermal-suppression period has increased by 0.8–3.1 d yr^−1^ in major agricultural regions such as eastern Europe (Fig. 4b), Indo-China Peninsula (Fig. 4c), the North China Plain (Fig. 4e) as well as savannas in Africa and the southeastern Amazon Basin (Fig. S17), and boreal forests in central Siberia (Fig. 4d). Other high-latitude boreal forests, however, have not changed significantly, indicating that they still have a considerable margin of safety for future warming (Fig. 4a) and even in the absence of thermal acclimation. These geographical differences may again reflect that for locations with higher interannual variability in temperature (e.g. more poleward), background warming may be less impactful. It is noted that the number of high-temperature limited days based on air temperature was comparable to the number based on canopy temperature (Figs S16 and S18a), but its trends were much weaker (Figs 4 and S18b).

Our data-led investigations have revealed the potential of vegetation to acclimate by quantifying the time-evolving values of ${T}^{\rm can}_{\rm opt} $. Such knowledge of ${T}^{\rm can}_{\rm opt} $ changes allows us to determine, by region, the extent to which acclimation capability mitigates the increases in temperatures that would otherwise reduce vegetation productivity. However, we found that thermal acclimation played only a limited role in alleviating heat stress (Fig. S19). Even after accounting for acclimation, 58% of global vegetated areas still experience a significant increase in thermal events (only slightly lower than 64% without acclimation). In tropical rainforests, widespread significant acclimation provides notable mitigation, with the annual rate reduction approaching 2 d yr^−1^ (Fig. 4f–h). Nevertheless, despite this buffering effect, the overall frequency of thermal limitations continues to rise rapidly, indicating that even substantial physiological adjustments are being outpaced by the rate of warming in these critical ecosystems. In contrast, across many other regions, including major agricultural areas, acclimation has only slightly reduced the rate of increase in days of vegetation productivity suppression (Fig. 4b–e). Together, these findings imply that the inherent adaptive capacity of vegetation in these areas was insufficient, leaving ecosystems still at considerable risk under the current pace of human-driven warming. Additionally, these conclusions are highly robust across multiple productivity proxies (Figs S20–S22).

## DISCUSSION AND CONCLUSIONS

Our data-driven findings highlight the escalating threat of thermal stress on vegetation due to human-induced climate change. Such stresses occur particularly when temperatures exceed ${T}^{\rm can}_{\rm opt} $, as above this value, productivity is rapidly suppressed. Under the initial assumption of invariance of ${T}^{\rm can}_{\rm opt} $, areas experiencing potential thermal limitations have expanded substantially, from 3.0 billion to a more recent coverage of 4.7 billion ha, comparable to the size of two Amazon rainforests (Fig. 3). Simultaneously, the frequency of thermally limiting events (i.e. when canopy temperature exceeds ${T}^{\rm can}_{\rm opt} $) has risen sharply, particularly in tropical forests and many agricultural regions (Fig. 4). In particular, the rate of intensification of heat stress over the recent period of 2018–2024 has far exceeded previous records (Figs 3 and 4), consistent with the increasing observed frequency and scope of extreme heatwaves in recent years [5,15,16]. Continuation of these trends would pose a serious threat to the global terrestrial carbon sink, future carbon-neutral targets and the overall health of land ecosystems and crop production [53].

We mainly base our assessment of changes in thermal stress on satellite-derived canopy temperature, as this more accurately represents the thermal regimes experienced by vegetation [30,32] (Fig. 1). Our analysis reveals a more rapidly escalating heat stress than the calculated changes based on air temperatures (Figs 3 and 4). This finding suggests that previous evaluations, instead of undertaking calculations with air temperatures only [8,14,54], while making valuable contributions to understanding, may have underestimated the thermal risks to vegetation. Recent studies have increasingly shown that the rate of warming under climate change at the top of a canopy may outpace the increase in air temperature due to changes in the physiological responses of the vegetation and radiation balance of the canopy [13,36]. Additional environmental stresses, such as water scarcity and reduced wind speeds, may further accelerate increases in canopy temperature [30,55,56]. These differences account for our larger increasing trends in exposure to high temperatures when based on canopy temperature, to which vegetation responses are more tightly linked.

By extending our analyses to include temporal variation in ${T}^{\rm can}_{\rm opt} $, we identified a thermal acclimation capability for many locations. Again, we suggest that our findings derived from canopy temperature, rather than air temperature, are likely to be more accurate, due to their ability to capture the vegetation responses more directly to environmental changes [30,32]. We found that over the past 22 years, the optimal canopy temperature has increased significantly in ∼19% of global vegetated regions, with these increases distributed across the planet (Fig. 2b). The primary drivers of this increase include elevated CO_2_ concentrations [10,57], physiological and morphological adjustments in plants [7,43,58], and shifts in community composition favouring heat-tolerant species [59–61]. In photosynthesis models, elevated CO_2_ concentrations can interact with a temperature response function such that the combined response simulates increases in ${T}^{\rm can}_{\rm opt} $. Alternatively, model parameters that might have previously been considered invariant may themselves adjust to raised temperatures (i.e. acclimate), a concept tested at a range of locations using eddy covariance measurements [62]. Our integrated framework merges independent strands of Earth Observation data to provide a globally complete assessment of ecosystem thermal acclimation. This advancement is of significant utility for the calibration and refinement of the land-surface components within Earth System Models (ESMs), which currently struggle to represent dynamic physiological adjustments. Moving beyond previous assessments that focused solely on the existence of acclimation [21,22], we evaluate the extent to which these physiological shifts actually mitigate intensifying thermal stress.

Our findings of thermal acclimation suggest that vegetation changes have slowed the rate of expansion and the increase in frequency of thermal suppression of vegetation productivity in many locations (Figs 3 and 4), thus mitigating adverse warming impacts on photosynthesis and the carbon cycle. This regulatory capacity of vegetation, however, is limited and cannot fully negate the detrimental trends associated with warming. Regions with intensified thermal limitations and limited capacity to acclimate, and so having substantial rises in thermal stress (e.g. major agricultural regions shown in Fig. 4), should be prioritized for greater attention and potentially specific targeted land management strategies in the future. In addition to *T*_opt_, recent evidence [63] suggests that the breadth of the photosynthetic temperature-response curve also significantly influences how vegetation responds to warming, which represents an important complementary dimension of thermal acclimation that warrants systematic investigation in future research.

While satellite-derived observations offer more physiologically relevant insights than air temperature [13,32], current thermal products primarily sample the radiometric temperature of the upper canopy and therefore cannot fully resolve thermal variation throughout the entire canopy profile [29]. Although the upper canopy accounts for the dominant proportion of total canopy photosynthesis [64], sub-canopy layers also contribute to productivity by utilizing diffuse and scattered light [65]. Site-specific research has shown, as expected, that the top of a canopy is exposed to the highest levels of solar radiation [64], whereas sub-canopy leaves are mostly shielded by the ‘parasol effect’ [66], creating microclimate buffering [35] with the potential to lower leaf temperature. The magnitude of these effects within canopies may vary across forest types and climatic zones, yielding vegetation responses to climate change that are more complex than those at the top of the canopy alone [64]. Hence, our main caveat is that while the approach presented here does provide robust information on the likely overall response of vegetation to thermal stress, it may be insufficient to fully characterize the response of the full vertical temperature profile or the thermal response of every canopy stratum in a warming world [29,66]. Further measurements of vertical foliar temperature profiles across diverse biomes are essential to bridge the gap between satellite measurements and the metabolic response of the entire canopy [66].

In addition, although we applied strict quality control at 1-km resolution to preferentially retain vegetation-dominated pixels, satellite-derived *T*_can_ may still be affected by soil background, mixed pixels, and vegetation clumping, particularly in sparsely vegetated ecosystems such as savannas. These effects likely contribute to the small but systematic deviations (Fig. S23) between satellite-derived and eddy-covariance (EC)-based *T*_can_ observed in some sparse ecosystems. Encouragingly, validation against site-scale aerodynamic canopy temperature estimates derived from EC observations showed generally good agreement (Figs S24 and S25), supporting the use of satellite-derived *T*_can_ as a meaningful proxy for ecosystem-scale canopy thermal conditions. Future work could further reduce these uncertainties by combining higher-resolution thermal observations with improved methods to separate canopy and soil thermal signals and to better resolve within-canopy temperature gradients.

Another critical frontier is the explicit incorporation of leaf temperature (*T*_leaf_). While our canopy-based framework represents vegetation thermal conditions more accurately than air temperature [30,32], *T*_leaf_ remains the most physiologically direct driver of photosynthesis. Recent evidence indicates that the divergence between *T*_can_ and *T*_leaf_ can be substantial in certain ecosystems [28], implying that canopy-integrated estimates may represent a conservative baseline for leaf-level heat stress. However, global assessments are currently hindered by the scarcity of long-term *T*_leaf_ observations [28,67,68] and the fact that most ESMs do not yet provide standardized diagnostics for both *T*_can_ and *T*_leaf_. Consequently, our analysis establishes a necessary canopy-scale benchmark, providing a foundation upon which future studies can build as sophisticated land-surface representations and comprehensive *T*_leaf_ monitoring networks become available.

Our study presents a novel, observation-led assessment of the recent response of photosynthesis to temperature and changes in thermal limitations, providing valuable guidance for ecosystem management in response to human-induced global warming. By capitalizing on simultaneous satellite measurements of canopy temperature and vegetation productivity, our analysis achieves nearly global coverage, capturing the heterogeneous ways ecosystems respond to heat stress. As climate warming intensifies, the challenges posed by thermal limitations on vegetation will become more acute, requiring models that can accurately simulate these impacts to inform societal adaptation. Despite the incorporation of nonlinear temperature-productivity responses, current global vegetation models rely primarily on air temperature [69,70] or utilize overly simplistic representations of canopy temperature [71] as the thermal forcing, which limits their fidelity in simulating actual photosynthetic performance. Furthermore, mechanistic representations of thermal acclimation are frequently omitted or highly simplified [72,73], with existing parameterizations often constrained by air temperature forcing rather than the more physiologically relevant leaf or canopy temperature. A significant hurdle for the modelling community is that *T*_can_ is not yet a standardized diagnostic variable in most ESMs, which precludes robust multi-model comparisons of thermal optima. Our analysis directly addresses these gaps, providing a blueprint for using satellite-derived metrics to constrain advanced land-surface model configurations. Moving forward, integrating these satellite insights with *in situ* data on within-canopy temperature profiles will be essential to refining our predictive capacity and ensuring the resilience of the global carbon sink.

## METHODS

### Canopy temperature

We derived global canopy temperature (*T*_can_) from the MODIS MYD21A1D land-surface temperature product at 1-km daily resolution for 2003–24 [74]. Because land-surface temperature approximates *T*_can_ in densely vegetated and homogeneous pixels, we retained vegetation-dominated pixels as canopy signals after excluding non-vegetated and mixed pixels. Specifically, we used the maximum–minimum apparent emissivity difference threshold (MMD < 0.03) to reduce mixed-pixel and soil-background contamination [13,75] together with an annual mean NDVI > 0.1 mask to remove water bodies and sparsely vegetated areas [8,76]. The Aqua overpass time, around 13:30 local time, is close to the timing of peak daytime canopy temperature, allowing *T*_can_ to represent maximum thermal exposure. Missing daily values caused by cloud contamination or retrieval errors were gap-filled using a 10-day moving average and, where necessary, long-term means; years and grid cells with excessive missing data were excluded. Satellite-based *T*_can_ was independently evaluated against canopy temperature estimated from eddy-covariance observations using an aerodynamic resistance approach [77,78], showing robust agreement across temporal aggregation scales (Figs S23–S25).

### Gross primary productivity and climate data

GPP, used as the primary indicator of ecosystem photosynthesis, was obtained from the MODIS MYD17A2HGP v6.1 product at 500-m and 8-day resolution [79]. We retained only good-quality and positive GPP values, then aggregated them to 1 km using area-weighted averaging to match the *T*_can_ dataset. The same MMD and NDVI filters were applied to ensure consistency between GPP and *T*_can_ observations. Growing-season months were derived from GIMMS leaf-area-index phenology and refined by excluding frozen periods [80]. To evaluate robustness, we further used three independent satellite-based proxies of photosynthetic activity: near-infrared reflectance of vegetation (NIRv) [39], NIRv multiplied by photosynthetically active radiation (NIRvP) [40], and contiguous solar-induced chlorophyll fluorescence (CSIF) [41]. Climate variables included daily maximum air temperature from ERA5-Land [81], precipitation from the Global Precipitation Climatology Project [82], with independent validation performed using Climatic Research Unit and Japanese Reanalysis [83,84] (Fig. S26). Daily maximum air temperature was emphasized because peak daytime heat is most directly linked to thermal limitation of photosynthesis.

### Optimal temperature for photosynthesis

The photosynthetic optimal temperature (*T*_opt_) was estimated from the response of GPP to canopy or air temperature, with NIR_V_, NIR_V_P, and CSIF used for validation. For each 0.25° window, all native-resolution (e.g. 1-km) pixels and time steps were pooled to derive a single *T*_opt_ value, thereby retaining sub-grid variability while improving statistical robustness. Daily *T*_can_ was first averaged to the 8-day resolution of GPP. GPP observations were then grouped into dynamic temperature bins, with bin widths adjusted according to local temperature variability. Within each bin, the median of the five highest GPP values was used to represent near-unconstrained photosynthetic performance, minimizing the influence of other limiting factors such as cloudiness, radiation limitation, or drought. A running average across three adjacent bins was then applied, and *T*_opt_ was identified as the temperature corresponding to the maximum of the smoothed response curve. *T*_opt_ was estimated over the full 2003–24 period for spatial analyses and annually to assess temporal shifts and thermal acclimation. Sensitivity tests using top-three, top-seven, and 90th-percentile [8] approaches yielded consistent results (Figs S27 and S28).

### Thermal acclimation

Thermal acclimation was defined as the temporal adjustment of *T*_opt_ to interannual variation in heat exposure [22]. For each grid cell, we regressed annual *T*_opt_ against annual maximum temperature (*T*_max_) over the 22-year record, with the slope ∂*T*_opt_/∂*T*_max_ representing the magnitude of acclimation. *T*_max_ was used because it better captures exposure to high-temperature conditions that constrain photosynthesis than growing-season mean temperature. Grid cells without a significant *T*_opt_-*T*_max_ relationship (*P*-value ≥ 0.05) were considered to lack detectable thermal acclimation and were assigned a fixed long-term *T*_opt_. Where significant acclimation was detected, annual *T*_opt_ was dynamically adjusted according to the local *T*_opt_-*T*_max_ relationship, isolating the temperature-driven component of *T*_opt_ variation from other confounding influences.

### Supra-optimal thermal stress

We assessed supra-optimal thermal stress from both the extent and frequency of temperatures exceeding *T*_opt_. The spatial extent of thermal limitation was defined as the area where growing-season mean temperature *T*_gs_ exceeded *T*_opt_. Negative values of *T*_gs_-*T*_opt_ indicated a remaining thermal safety margin, whereas positive values indicated that vegetation was already operating above its photosynthetic optimum and experiencing thermal suppression. We further calculated the annual number of days when the daily maximum temperature exceeded *T*_opt_, using both fixed and acclimation-adjusted *T*_opt_ values. Temporal trends in these heat-stress days were estimated using least-squares linear regression globally and across key carbon-sink and food-producing regions. Further details on datasets, preprocessing procedures, validation methods, *T*_opt_ estimation, thermal acclimation, and thermal-risk assessment are provided in the Supplementary Information.

## Supplementary Material

nwag347_Supplemental_File

## Data Availability

All observational and reanalysis datasets used in this study are publicly available. MODIS data products (including LST, GPP, NIR_T_, and NDVI) can be obtained from NASA’s Land Processes Distributed Active Archive Center (LP DAAC: https://lpdaac.usgs.gov). The FLUXNET2015 dataset used for validation is available at https://fluxnet.org/data/fluxnet2015-dataset/. The incident photosynthetically active radiation (PAR) data from the Global Land Surface Satellite (GLASS) are available at https://www.glass.hku.hk/. The ERA5-Land reanalysis data and the GPCP v2.3 monthly precipitation product are accessible through the Copernicus Climate Change Service (C3S) Climate Data Store (https://cds.climate.copernicus.eu). The CRU-JRA reanalysis products are accessible via the CEDA Archive website (https://archive.ceda.ac.uk). The CSIF dataset can be accessed through Figshare (https://doi.org/10.6084/m9.figshare.6387494). The canopy temperature dataset and primary results generated in this study are archived on Figshare: https://doi.org/10.6084/m9.figshare.31156939. All computer codes used for processing and analysing the data are available through Figshare: https://doi.org/10.6084/m9.figshare.31156939.
