# Supplementary material for: Increasingly severe thermal stresses on global photosynthesis using insights from observations of canopy temperature
Source: Natl Sci Rev. 2026 Jun 22;13(13):nwag347. doi: 10.1093/nsr/nwag347 (PMC13352433; doi:10.1093/nsr/nwag347)
Supplement: nwag347_Supplemental_File [file nwag347_supplemental_file.docx]

**Supplementary Information for**

**Increasingly severe thermal limitations on global** **photosynthesis using insights from observations of canopy temperature**

Tianbo Pan^1^, Hao Xu^1,2*^, Chris Huntingford^3^, Shuchang Tang^1^,

Kai Wang^1^, Josep Peñuelas^4,5^, Shilong Piao^1*^

^1^ Sino-French Institute for Earth System Science, College of Urban and Environmental Sciences, Peking University, Beijing 100871, China.

^2^ International Institute for Applied Systems Analysis, Laxenburg 2361, Austria

^3^ UK Centre for Ecology and Hydrology, Wallingford OX10 8BB, UK

^4^ CREAF, Catalonia, Barcelona 08193, Spain

^5^ CSIC, Global Ecology Unit CREAF-CSIC-UAB, Barcelona 08193, Spain

*Corresponding author: Shilong Piao ([slpiao@pku.edu.cn](mailto:slpiao@pku.edu.cn)), Hao Xu ([xuhao@iiasa.ac.at](mailto:xuhao@iiasa.ac.at))

**The file includes:**

**Supplementary Text**

**Supplementary Figure S1-S31**

**Reference**

**Supplementary Text**

**Canopy temperature**

Canopy temperature (*T*_can_), defined as the average temperature of multiple foliar assemblages aggregated in whole crowns, strongly impacts exchanges of water, energy, and carbon between an ecosystem and atmosphere [1,2]. Canopy temperature and land-surface temperature (LST) are effectively equivalent in densely vegetated and relatively homogeneous areas, so we derived canopy temperatures around the globe from satellite observations using a straightforward method that defines LST for pixels of vegetation-dominated pixels, after excluding non-vegetated and mixed pixels, as the canopy temperature. To identify such pixels, we primarily used the maximum–minimum apparent emissivity difference (MMD) as a screening metric [3] (MMD<0.03). Previous studies have shown that MMD provides a more conservative estimate of complete canopy cover than land-cover classifications and is effective in reducing contamination from mixed pixels and soil background [4]. Because MMD alone cannot reliably distinguish vegetation from water, we additionally applied a broad vegetation mask based on the normalized difference vegetation index (annual mean NDVI > 0.1) to exclude water bodies and areas with very sparse vegetation. To better account for the spatial heterogeneity of natural vegetation, all subsequent analyses of optimal temperature were conducted at the native 1-km resolution, thereby preserving fine-scale spatial information and minimizing the effects of landscape patchiness. Even with these filters, the derived *T*_can_ should be interpreted as an ecosystem-scale radiometric signal of the vegetation canopy, with greatest sensitivity to the upper canopy rather than the full vertical temperature profile.

Remotely sensed LST data were derived from the Moderate Resolution Imaging Spectroradiometer (MODIS) (MYD21A1D version 6) product, which has provided a spatial resolution of 1 km and a temporal resolution of 1-day from 2002 to 2024 [5]. We excluded 2002 because the LST record begins in early July 2002 and therefore does not provide complete annual coverage. We chose this product for several reasons. Firstly, it retrieves LST using the Temperature/Emissivity Separation algorithm, which provides MMD as a by-product and is also widely used in the two most recent remote-sensing sensors: Visible Infrared Imaging Radiometer Suite (VIIRS) and the Ecosystem Spaceborne Thermal Radiometer Experiment on Space Station (ECOSTRESS). Secondly, it offers more than 20 years of continuous observations, which is much longer than those of the other two sensors, making it highly suitable for decadal analyses. This product also incorporates an improved scheme for correcting the scaling of atmospheric water vapor to stabilize the retrieval during very warm and humid conditions. The time of the overpass of the Aqua satellite (13:30) is similar to the time of maximum canopy temperature (14:00–16:00) [6,7], so we assumed that the canopy temperatures obtained from this product would be comparable to the daily maximum canopy temperature. Auxiliary NDVI data from 2000 to 2024 were obtained from the MOD13A2 v6 product with a spatial resolution of 1 km and a temporal resolution of 16 days [8]. Pixels with a mean annual NDVI>0.1 were defined as vegetated areas. This threshold has been widely adopted in previous large-scale vegetation studies [6], designed to exclude bare soil while retaining all major vegetation types. To assess the sensitivity of this criterion, we repeated the analysis using alternative NDVI thresholds of 0.05 and 0.15, which yielded consistent results (Fig. S29).

To validate the robustness of the satellite-based *T*_can_, we independently calculated *T*_can_ from eddy covariance (EC) measurements using a widely applied aerodynamic approach [9,10] (*T*_can_aefro_; Eq.1; Figs. S23-S25). The calculation was based on hourly flux data from the FLUXNET2015 dataset [11], using observations at 13:30 local time to match the MODIS overpass:

$$T_{can\_aero}=\left( \frac{u}{\left( u^{*} \right)^{2}}+6.2{u^{*}}^{-\frac{2}{3}} \right)\times\left( \frac{H}{c_{p}\rho_{a}} \right)+T_{a} (Eq.1)$$

where *u* is the horizontal wind speed (m s^-1^), *u*^*^ is the friction velocity (m s^-1^), *H* is the sensible heat flux (W m^-2^), *c*_p_ is the specific heat capacity of air (=29.3 J mol^-1^ K^-1^), *ρ*_a_ is the density of wet air (kg m^3^), and *T*_a_ is the air temperature (K).

The density of moist air was calculated as follows (Eq.2):

$$\rho_{a}=\frac{p-e}{R_{d}T_{a}}+\frac{e}{R_{v}T_{a}} (Eq.2)$$

where *p* is atmospheric pressure (Pa), *e* is the water vapour pressure (Pa), *R*_d_ is the specific gas constant for dry air (=287.05 J kg^-1^ K^-1^), and *R*_v_ is the specific gas constant for water vapour (=461.5 J kg^-1^ K^-1^).

As an independent evaluation, we compared the EC-derived *T*_can_ with satellite-based *T*_can_ during the growing season. Our results showed that the two estimates were highly consistent across temporal scales (1-, 4-, 8-, and 16-day) when pooled across all sites (Fig. S24), and this consistency was also evident when comparing site-level averages (Fig. S25).

In addition, to ensure data completeness, we applied quality control and gap-filling to the daily canopy temperature time series to address missing values caused by, for example, poor meteorological conditions and errors in the satellite equipment. If more than 80% of the data was missing for a given year, that year was excluded from the calculations of interannual variability. Grid cells with more than five missing years over the 22 years were excluded from the analysis. We used a moving-average method with a 10-day window to fill missing values for the remaining data, and any residual gaps were filled using the long-term mean. This gap-filling step is necessary because several key metrics (e.g., the frequency of thermal stress) depend on temporal continuity; neglecting missing days can lead to a systematic underestimation of heat-stress days (Fig. S30). To further evaluate whether gap-filling could degrade data quality or introduce artefacts, we compared the gap-filled product with EC-derived aerodynamic canopy temperature and found consistently high agreement across temporal aggregation scales (Fig. S31), comparable to that obtained using original data (Fig. S24).

**Gross primary productivity and growing-season data**

The gross primary productivity (GPP) represents the total amount of carbon fixed through photosynthesis by vegetation and serves as a key indicator of ecosystem function [12,13]. Here, we used satellite-derived GPP to estimate the optimal canopy and air temperatures for global photosynthesis at the ecosystem scale. The GPP data were obtained from the MYD17A2HGP v6.1 product, which is based on the light-use efficiency theory and has a temporal resolution of 8 days and a spatial resolution of 500m. The MYD17A2HGF product, an improved version of MOD17, eliminates poor-quality inputs from the 8-day LAI and FPAR using pixel-level quality control [14]. To ensure data reliability, we applied the MODLAND_QC layer and retained only pixels where Bit 0 was 0. According to the product documentation, this flag signifies Good Quality, indicating that GPP was produced by the main algorithm rather than by a backup or fill procedure. The filtered GPP data were then aggregated to 1 km using an area-weighted averaging approach to match the spatial resolution of the canopy temperature dataset. To maintain consistency between datasets, we further applied MMD and NDVI indicators to identify pixels with pure vegetation cover. Only positive GPP values were retained for subsequent analyses. We also derived information on the growing-season months from [15], which was determined from the GIMMS dataset for the leaf area index using a Savitzky–Golay filter and then refined by excluding the ground-freeze period identified by the data records for the freeze/thaw Earth system.

**Additional GPP proxies**

To validate the robustness of our results, we additionally used three satellite-based proxies of photosynthetic activity: the near-infrared reflectance of vegetation (NIR_V_) [16], the product of NIR_V_ and photosynthetically active radiation (NIR_V_P) [17], and the contiguous solar-induced chlorophyll fluorescence (CSIF) [18].

NIR_V_, the product of the total near-infrared reflectance (NIR_T_) and normalized difference vegetation index (NDVI), better represents observed ecosystem photosynthesis. NIR_V_ effectively excludes non-vegetative influences in mixed pixels and well eliminates the cofounding effects of background brightness, foliar area, and the distribution of photosynthetic capacity with depth in canopies [16]. As a proxy of photosynthesis, NIR_V_ has been suggested to be more strongly correlated with GPP than NDVI at both spatial and temporal scales [19,20]. Following previous studies [6], we calculated 16-day NIR_V_ for 2003–2024 as the product of MODIS 16-day NIR_T_ and MODIS 16-day NDVI, both obtained from the MOD13A2 v2 product with a resolution of 1 km. For quality control, we used the VI Quality (MODLAND QA Bits) layer and retained only pixels where Bits 0-1 were 00, which signifies VI was produced with good quality according to the product documentation. Similar to the GPP processing, we then applied the MMD and NDVI indicators to remove non-vegetation and mixed pixels. Only positive values were used in subsequent analysis.

However, NIR_V_ does not account for any radiation availability (e.g., PAR) or physiological information (e.g., light-use efficiency), which limits its capacity to fully represent the photosynthetic [17]. To address this gap, we further employed the product of NIR_V_ and incident photosynthetically active radiation (NIR_V_P), which integrates both canopy structural and radiative components. Recent studies have shown that NIR_V_P provides a more comprehensive representation of photosynthesis activity and exhibits stronger correlations with GPP and SIF than NIR_V_ alone [17]. Daily PAR data were obtained from the GLASS04B01.V42 product [21] with a spatial resolution of 0.05°. To ensure spatiotemporal consistency, we aggregated NIR_V_ to 0.05° using an area-weighted averaging approach and composited daily PAR values into 16-day means. The resulting NIR_V_P dataset thus has a spatial resolution of 0.05° and a temporal resolution of 16 days.

To complement NIR_V_- and NIR_V_P-based results, we additionally used satellite-based solar-induced chlorophyll fluorescence (SIF) data, which provide a direct indicator of photosynthetic activity and are closely correlated with GPP [18]. The SIF data were obtained from the contiguous solar-induced chlorophyll fluorescence (CSIF) dataset, which provides a 4-day temporal resolution from 2000 to 2023 and a spatial resolution of 0.05°. The CSIF dataset was generated by training a neural network with MODIS surface reflectance data and SIF measurements from the Orbiting Carbon Observatory-2 (OCO-2). This approach effectively overcomes limitations of traditional SIF products, including their coarse spatiotemporal resolution, high retrieval uncertainty, and inconsistent measurement footprints.

**Climatic data**

The gridded data for air temperature were derived from the ECMWF Reanalysis v5 (ERA5-Land) product, which has a spatial resolution of 0.1° and a daily temporal resolution, covering years from 1950 to 2024 [22]. We included results based on air temperature for comparison in our analysis to investigate the discrepancies between estimates of air temperature and observations of canopy temperature. Data for canopy temperature and GPP from MODIS represent the condition of canopies at the time of the daily maximum temperature, so we similarly focused our analysis on daily maximum air temperature, in which thermal limitations for vegetation first occur under continued warming. Daily mean temperature, which include both day- and night-time temperatures, was used only for validation (Fig. S2). We used the observation-based Global Precipitation Climatology Project (GPCP) product, which provides daily precipitation data from 1996 to 2023 with a resolution of 1° [23], due to large uncertainties in estimates of precipitation from reanalysis data. In addition, the CRUJRA reanalysis product [24,25] was used to independently validate our estimates of *T*air opt (Fig. S26).

**Optimal temperature for photosynthesis**

We estimated the optimal canopy temperature (*T*can opt) for ecosystem photosynthesis primarily from GPP data, with NIR_V_, NIR_V_P, and CSIF for validation. For each 0.25° window, a single *T*_opt_ value was calculated directly from all pixels at native resolution. In the case of GPP, each window’s *T*_opt_ was computed from the 30 × 30 pixels of 1-km GPP and *T*_can_ data. Compared to traditional approaches that first aggregate data before estimation, this method preserves high-resolution information and ensures robust estimates. The same window-based approach was applied to the other validated proxies, with the number of pixels per window varying according to native resolution. For air temperature, given its native resolution of 0.1°, *T*_opt_ was derived on a 0.5° window.

Specifically, we estimated *T*can opt by examining the temperature-response curve of GPP. To match the temporal resolution of the 8-day GPP data, daily *T*_can_ was first averaged over 8 days. *T*can opt was then extracted through a four-step procedure: (1) GPP time series over the entire monitoring period and the corresponding temperature data were grouped into dynamic temperature bins, with widths ranging from 0.25 °C to 1 °C depending on local temperature variability; (2) within each bin, the median of the five highest GPP values was used to represent the GPP response. This selection thereby eliminates the potential influences of other environmental constraints, such as cloud cover and drought, as the highest values are unlikely to have such additional limitations. The sensitive tests using the top three or top seven values (Fig. S27), as well as the 90^th^ percentile methods (Fig. S28) used in previous studies [6], yielded consistent results; (3) the running average method for every three temperature bins was applied to construct the temperature-response curve, which effectively removes outliers and smoothes the temperature-response curves for reliable *T*_opt_ identification; (4) *T*_opt_ was identified as the temperature corresponding to the peak of this response curve. If the maximum value occurs at the edges of the curve, *T*_opt_ may not exist for that pixel during the monitoring period. In this study, *T*_opt_ was first calculated over the entire monitoring period to examine its spatial distribution and relationship with the climatic background, and it was also computed on an annual interval to assess temporal changes and quantify the magnitude of thermal acclimation.

We investigated the relationship between *T*_opt_ (i.e., *T*can opt and *T*air opt) and climatic variables by averaging *T*_opt_ in the climatic space with mean annual intervals of 0.7°C averaged over the growing season (*T*air gs) and 70-mm intervals of mean annual precipitation (MAP) (Fig. 1c, d). For each MAP (or *T*air gs) interval, we calculated the apparent spatial sensitivity of *T*_opt_ in response to changes in *T*air gs (or MAP) using a bootstrapping method to ensure robustness and reproducibility of the result. We performed the linear regression analysis 1000 times by randomly selecting a subset of 80% of the samples from pairs of *T*_opt_ and *T*air gs (or MAP) within each MAP (or *T*air gs) interval. The means and standard deviations of the sensitivities of *T*can opt and *T*air opt to temperature and precipitation were subsequently estimated along the gradients of MAP and *T*air gs.

**Thermal acclimation**

Thermal acclimation, defined as the adjustment of vegetation to rising temperatures through physiological and morphological changes, can mitigate the adverse effects of heat stress on photosynthesis. In this study, thermal acclimation specifically refers to the temporal sensitivity of *T*_opt_ to heat stress at the same grid, distinct from its spatial dependence on temperature. Following previous studies [26], we quantified thermal acclimation by performing a Pearson regression between annual *T*_opt_ against annual maximum temperature (*T*_max_) over the last 22 years for each grid, with the regression slope (∂*T*_opt_/∂*T*_max_) representing its magnitude. *T*_max_ was used because it better captures exposure to high-temperature conditions that constrain photosynthesis and drive heat stress than growing-season mean temperature. Grids where the regression was not significant (*P*-value≥0.05) were considered to lack thermal acclimation.

To incorporate thermal acclimation into *T*_opt_, we adjusted the annual *T*_opt_ for each grid according to the magnitude and significance of acclimation. Specifically, in regions without significant thermal acclimation, interannual variations of *T*_opt_ were assumed to be driven by non-temperature factors, and *T*_opt_ was assigned as a constant value calculated over 2003-2024. In contrast, for regions with significant thermal acclimation, annual *T*_opt_ was dynamically adjusted using the linear relationship between annual *T*_max_ and the estimated acclimation magnitude, thereby isolating the temperature-driven component of *T*_opt_ variation and minimising the influence of other confounding factors. This approach provides a more realistic representation of vegetation’s adaptive thermal response and improves the accuracy of subsequent heat stress assessment.

**Supra-optimal thermal limitations**

Thermal limitations, defined as conditions where the temperature for vegetation growth exceeds *T*_opt_, have become increasingly widespread and frequent under global warming, potentially reducing terrestrial carbon uptake. In this study, we investigated both the extent and frequency of thermal limitations on global vegetation photosynthesis. Specifically, the extent of thermal limitation was assessed using the difference between *T*_opt_ and *T*_gs_. When *T*_opt_ was >*T*_gs_, the difference represented a safety margin for vegetation productivity, as further warming still had a positive effect on photosynthesis. Conversely, when *T*_gs_ was >*T*_opt_, the area was deemed already subject to thermal suppression, with the difference indicating the degree of thermal limitation. In addition to examining spatial patterns, we tracked interannual variations in thermally limited area using both fixed and acclimation-adjusted *T*_opt_ values (Fig. 3).

Furthermore, to quantify the frequency of thermal limitations, we calculated the number of days per year when the daily maximum temperature exceeds *T*_opt_. Our analysis mainly focused on the spatial distribution and temporal trends of these high-temperature days, with trends estimated using least squares linear regression. We further examined these temporal variations in seven key regions, which are either important carbon sinks or food-producing regions, and are therefore crucial for human society and the Earth system. These analyses were conducted using both a fixed *T*_opt_ (without acclimation) and a dynamically adjusted *T*_opt_ (with thermal acclimation) (Fig. 4), which could further contribute to understanding broader thermal stress patterns and influencing factors (e.g., El Niño events, extreme heatwave events).

**Supplementary Figures**


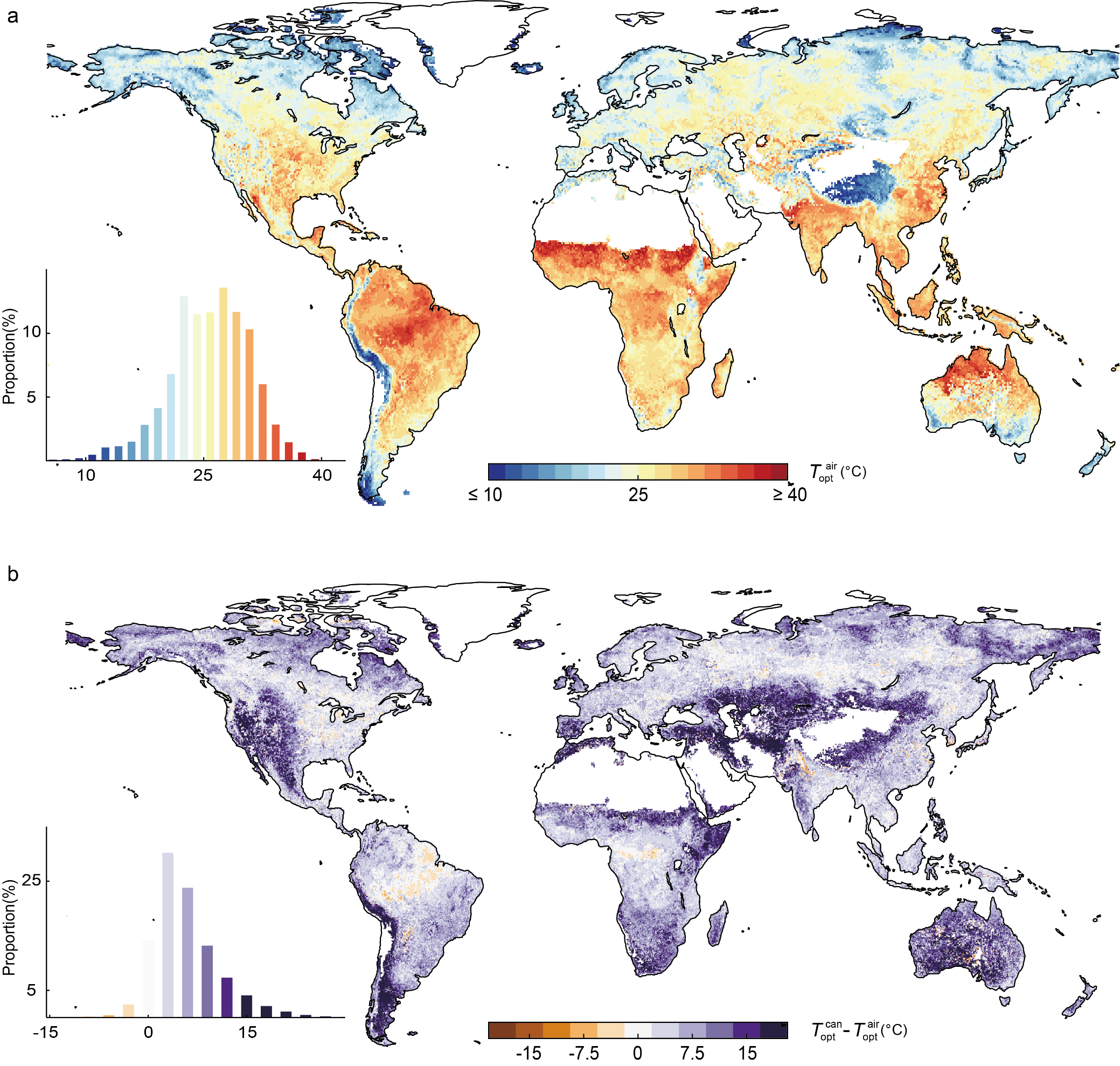


**Figure S1.** **Distributions of derived *Tair opt* values for vegetation productivity and the difference between *Tcan opt* and *Tair opt*. a**, Spatial distribution of *T*air opt for photosynthesis determined using ERA5-Land air temperature and MODIS GPP values from 2003 to 2024. Values are only presented for vegetated areas (i.e., for annual mean normalized difference vegetation index (NDVI) value larger than 0.1), where a value of *T*can opt is detected and where the growing season lasts longer than one month; otherwise, the location is blank (i.e., white). The inset histogram represents the areal proportion for different magnitudes of *Tair opt*. **b**, Distribution of the difference between *Tcan opt* and *Tair opt.* The inset histogram represents the areal proportion for magnitudes of differences.

**
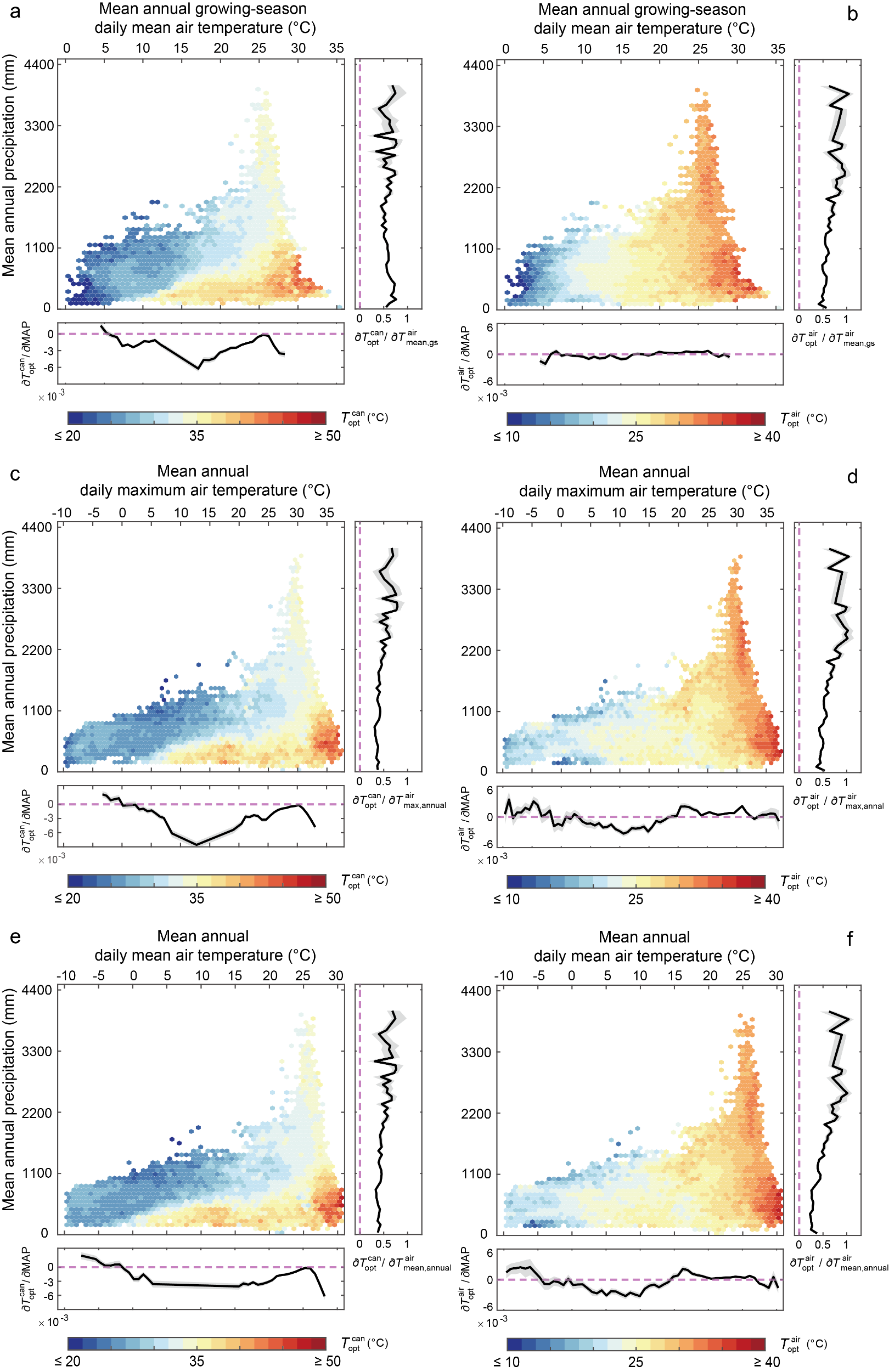
**

**Figure S2. *Tcan opt* and *Tair opt* in the temperature-precipitation space using different metrics. a,** The climatic dependence of *T*can opt on temperature and precipitation, along with relative sensitivities (side panels). Note the “*x*”-axis label and values are presented across the top of the main panels. Each climatic bin was defined by intervals of 0.7℃ of mean annual growing-season daily mean air temperature (*T*air mean, gs) and 70-mm intervals of mean annual precipitation (MAP), based on current climatic conditions averaged between 2003 and 2024. The solid line in the extra bottom (extra right) panel represents the sensitivity to temperature (precipitation) along the precipitation (temperature) gradient, calculated as the slope of the linear regression between *T*can opt and *T*air mean, gs (MAP) for a given level of precipitation (temperature). The shaded area indicates the standard deviation of the sensitivity (∂*T*can opt/∂*T*air mean, gs, or ∂*T*can opt/∂MAP) estimated by bootstrapping. **b,** Similar to **a**, but focuses on the optimum air temperature *T*air opt. **c, d,** and **e, f** are similar to **a, b**, but focus on the mean annual daily maximum air temperature (*T*air max, annual) and mean annual daily mean air temperature (*T*air mean, annual), respectively.


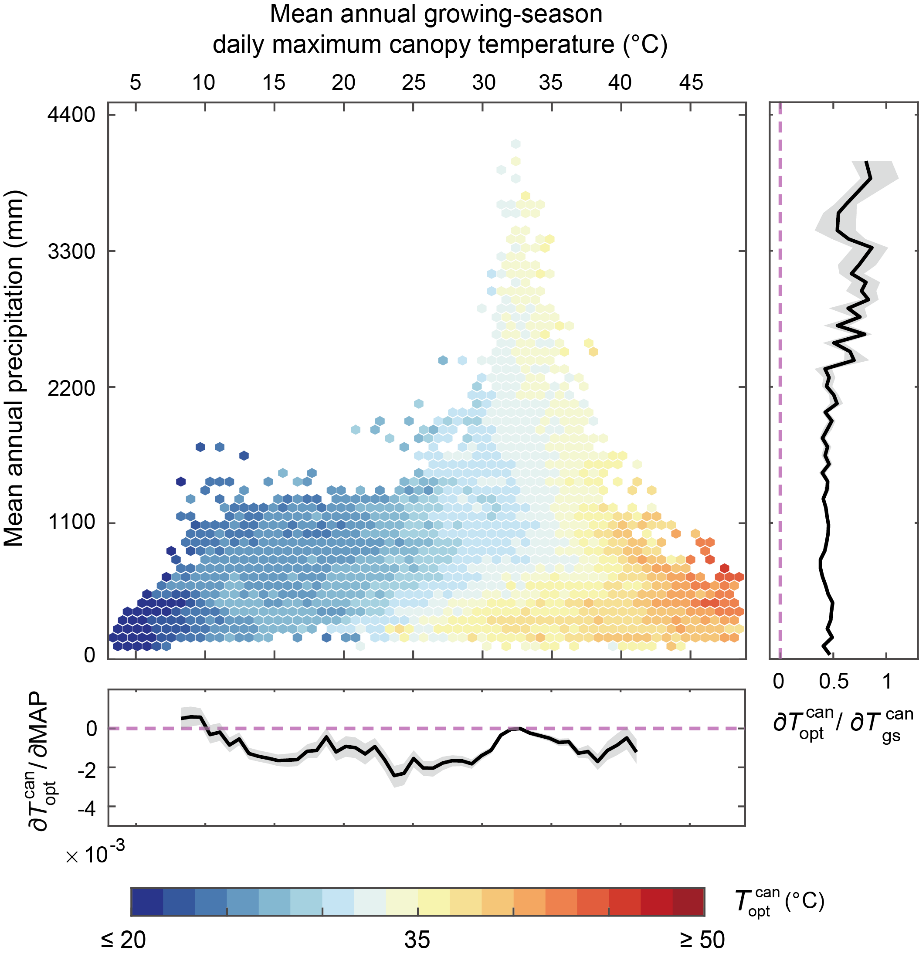


**Figure S3. Spatial sensitivity of *Tcan opt* to mean annual growing-season canopy temperature.** The climatic dependence of *T*can opt on temperature and precipitation, along with relative sensitivities (side panels). Each climatic bin was defined by intervals of 0.7℃ of mean annual growing-season daily maximum canopy temperature (*T*can gs) and 70-mm intervals of mean annual precipitation (MAP), based on current climatic conditions averaged between 2003 and 2024. The solid line in the extra bottom (extra right) panel represents the sensitivity to temperature (precipitation) along the precipitation (temperature) gradient, calculated as the slope of the linear regression between *T*can opt and *T*can gs (MAP) for a given level of precipitation (temperature). The shaded area indicates the standard deviation of the sensitivity (∂*T*can opt/∂*T*can gs, or ∂*T*can opt/∂MAP) estimated by bootstrapping.


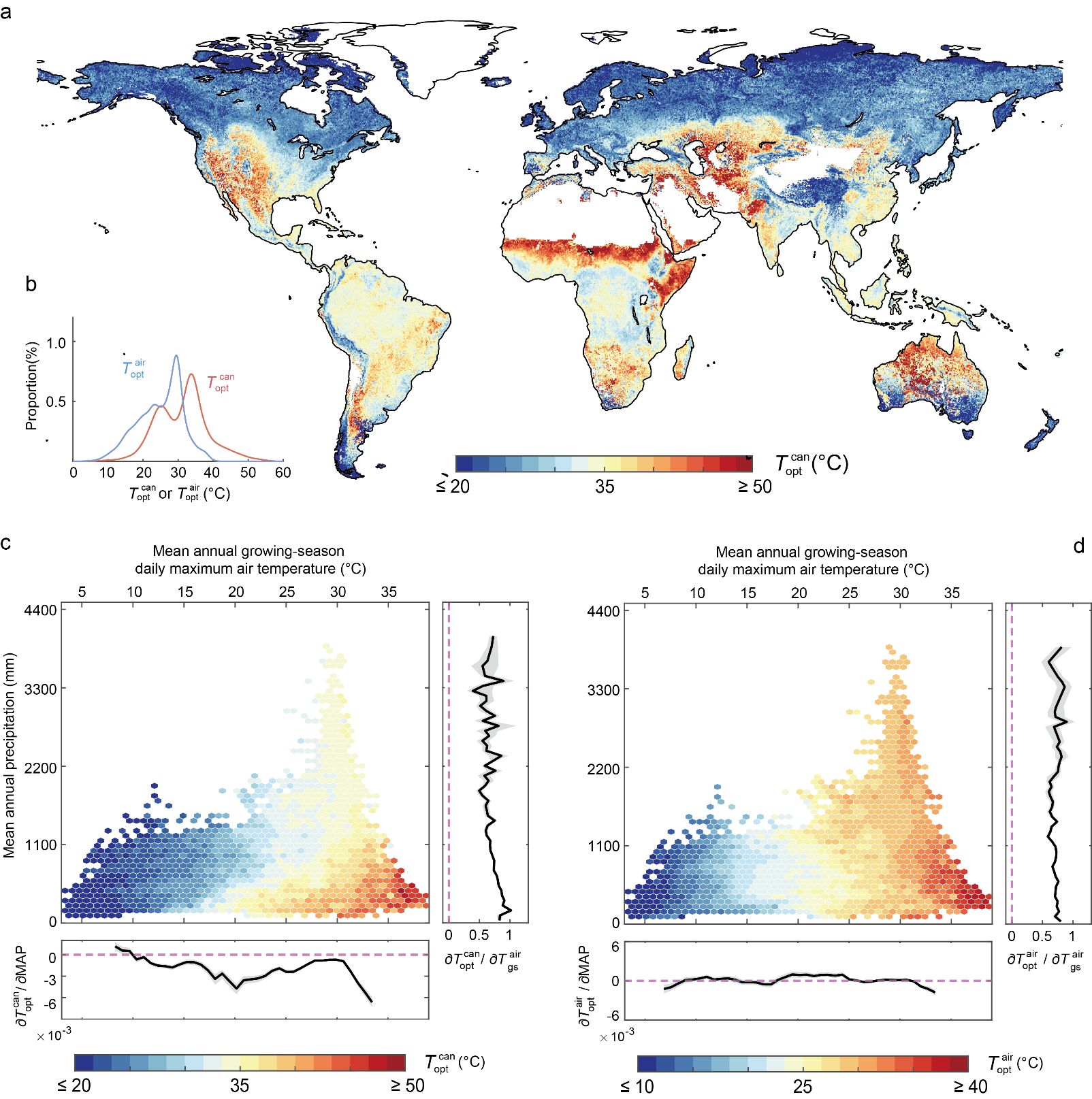


**Figure S4. Distributions of derived *T*can opt and *T*air opt values for vegetation productivity and their connection to background climate, based on the NIR_V_ proxy. a**, Spatial distribution of *T*can opt for photosynthesis determined using simultaneous canopy temperature and NIR_V_ values based on satellite observations from the Moderate Resolution Imaging Spectroradiometer (MODIS). The satellite data used covers the years 2003 to 2024 inclusive. Values are only presented for vegetated areas (i.e., for annual mean normalized difference vegetation index (NDVI) values larger than 0.1), where a value of *T*can opt is detected and where the growing season lasts longer than one month; otherwise, the location is blank (i.e., white). **b**, The red probability-density function (expressed as a percentage), represents the distribution of *T*can opt (as shown in panel **a**) based on the proportion of its actual area relative to the total vegetated area. The blue line represents the same distribution but for *T*air opt, with the detailed spatial pattern shown in Fig. S1a. **c**, The climatic dependence of *T*can opt on air temperature and precipitation, along with relative sensitivities (side panels). Note the “*x*”-axis label and values are presented across the top of the main panels **c** and **d**. Each climatic bin was defined by intervals of 0.7℃ of mean annual growing-season daily maximum air temperature (*T*air gs) and 70-mm intervals of mean annual precipitation (MAP), based on current climatic conditions averaged between 2003 and 2024. The solid line in the extra bottom (extra right) panel represents the sensitivity to temperature (precipitation) along the precipitation (temperature) gradient, calculated as the slope of the linear regression between *T*can opt and *T*air gs (MAP) for a given level of precipitation (temperature). The shaded area indicates the standard deviation of the sensitivity (∂*T*can opt/∂*T*air gs or ∂*T*can opt/∂MAP) estimated by bootstrapping. **d,** Similar to **c**, but instead makes all calculations of *T*_opt_ based on air temperature rather than canopy temperature, and therefore presents variations in *T*air opt.


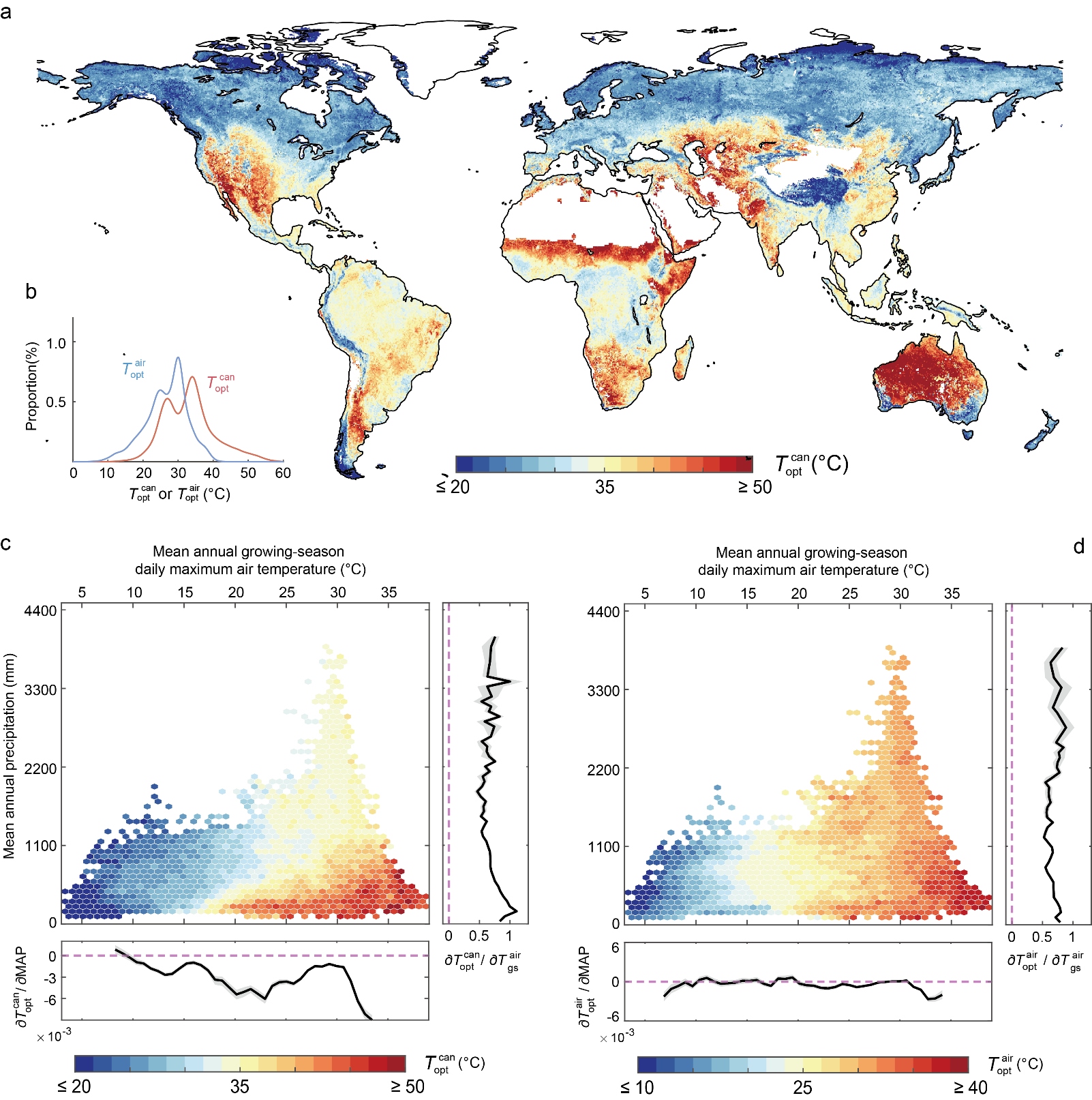


**Figure S5. Distributions of derived *T*can opt and *T*air opt values for vegetation productivity and their connection to background climate, based on the NIR_V_P proxy. a**, Spatial distribution of *T*can opt for photosynthesis determined using simultaneous canopy temperature and NIR_V_P values. The satellite data used covers the years 2003 to 2024 inclusive. Values are only presented for vegetated areas (i.e., for annual mean normalized difference vegetation index (NDVI) values larger than 0.1), where a value of *T*can opt is detected and where the growing season lasts longer than one month; otherwise, the location is blank (i.e., white). **b**, The red probability-density function (expressed as a percentage), represents the distribution of *T*can opt (as shown in panel **a**) based on the proportion of its actual area relative to the total vegetated area. The blue line represents the same distribution but for *T*air opt, with the detailed spatial pattern shown in Fig. S1a. **c**, The climatic dependence of *T*can opt on air temperature and precipitation, along with relative sensitivities (side panels). Note the “*x*”-axis label and values are presented across the top of the main panels **c** and **d**. Each climatic bin was defined by intervals of 0.7℃ of mean annual growing-season daily maximum air temperature (*T*air gs) and 70-mm intervals of mean annual precipitation (MAP), based on current climatic conditions averaged between 2003 and 2024. The solid line in the extra bottom (extra right) panel represents the sensitivity to temperature (precipitation) along the precipitation (temperature) gradient, calculated as the slope of the linear regression between *T*can opt and *T*air gs (MAP) for a given level of precipitation (temperature). The shaded area indicates the standard deviation of the sensitivity (∂*T*can opt/∂*T*air gs or ∂*T*can opt/∂MAP) estimated by bootstrapping. **d,** Similar to **c**, but instead makes all calculations of *T*_opt_ based on air temperature rather than canopy temperature, and therefore presents variations in *T*air opt.


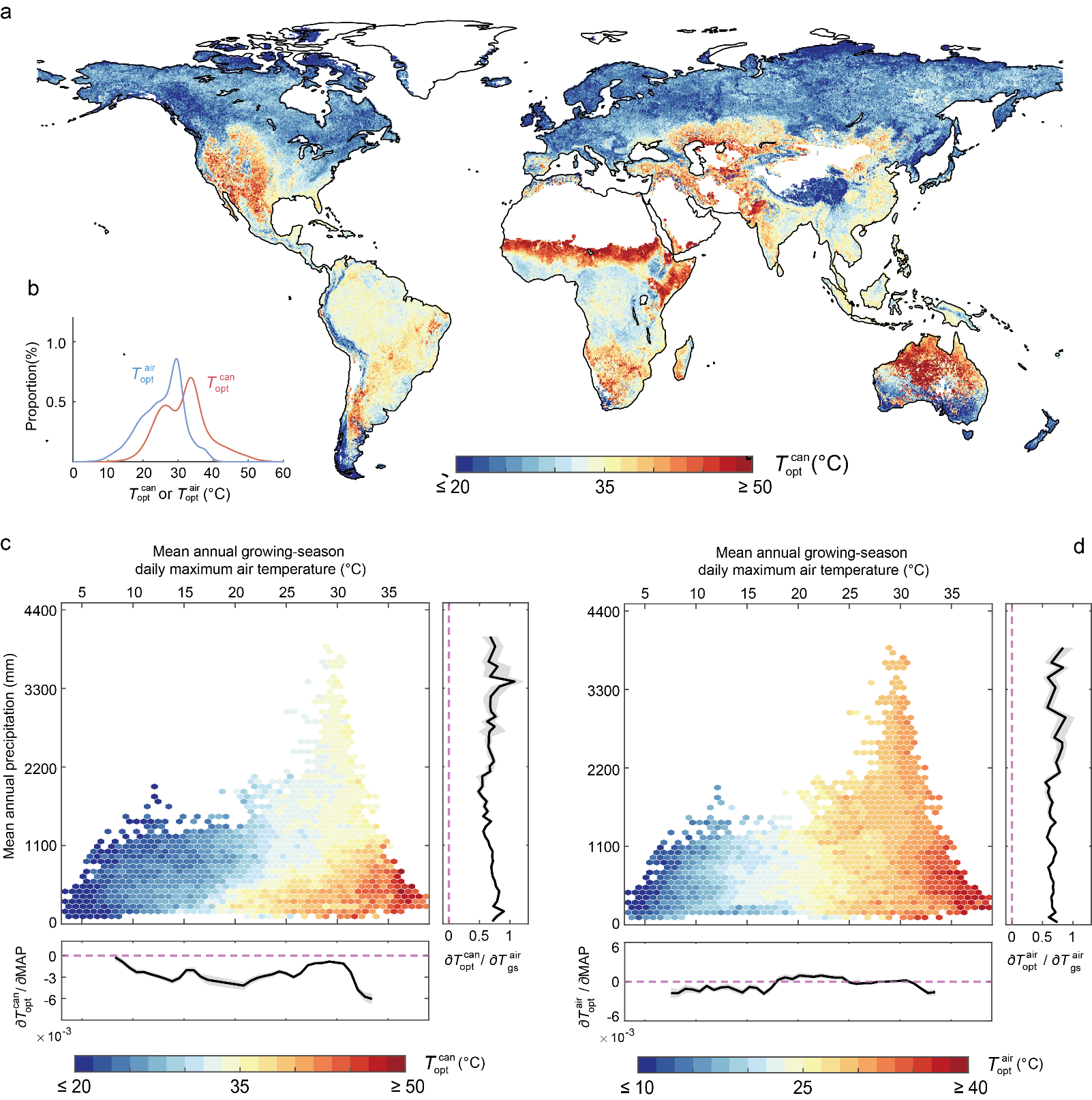


**Figure S6. Distributions of derived *T*can opt and *T*air opt values for vegetation productivity and their connection to background climate, based on the CSIF proxy. a**, Spatial distribution of *T*can opt for photosynthesis determined using simultaneous canopy temperature and CSIF values. The satellite data used covers the years 2003 to 2024 inclusive. Values are only presented for vegetated areas (i.e., for annual mean normalized difference vegetation index (NDVI) values larger than 0.1), where a value of *T*can opt is detected and where the growing season lasts longer than one month; otherwise, the location is blank (i.e., white). **b**, The red probability-density function (expressed as a percentage), represents the distribution of *T*can opt (as shown in panel **a**) based on the proportion of its actual area relative to the total vegetated area. The blue line represents the same distribution but for *T*air opt, with the detailed spatial pattern shown in Fig. S1a. **c**, The climatic dependence of *T*can opt on air temperature and precipitation, along with relative sensitivities (side panels). Note the “*x*”-axis label and values are presented across the top of the main panels **c** and **d**. Each climatic bin was defined by intervals of 0.7℃ of mean annual growing-season daily maximum air temperature (*T*air gs) and 70-mm intervals of mean annual precipitation (MAP), based on current climatic conditions averaged between 2003 and 2024. The solid line in the extra bottom (extra right) panel represents the sensitivity to temperature (precipitation) along the precipitation (temperature) gradient, calculated as the slope of the linear regression between *T*can opt and *T*air gs (MAP) for a given level of precipitation (temperature). The shaded area indicates the standard deviation of the sensitivity (∂*T*can opt/∂*T*air gs or ∂*T*can opt/∂MAP) estimated by bootstrapping. **d,** Similar to **c**, but instead makes all calculations of *T*_opt_ based on air temperature rather than canopy temperature, and therefore presents variations in *T*air opt.


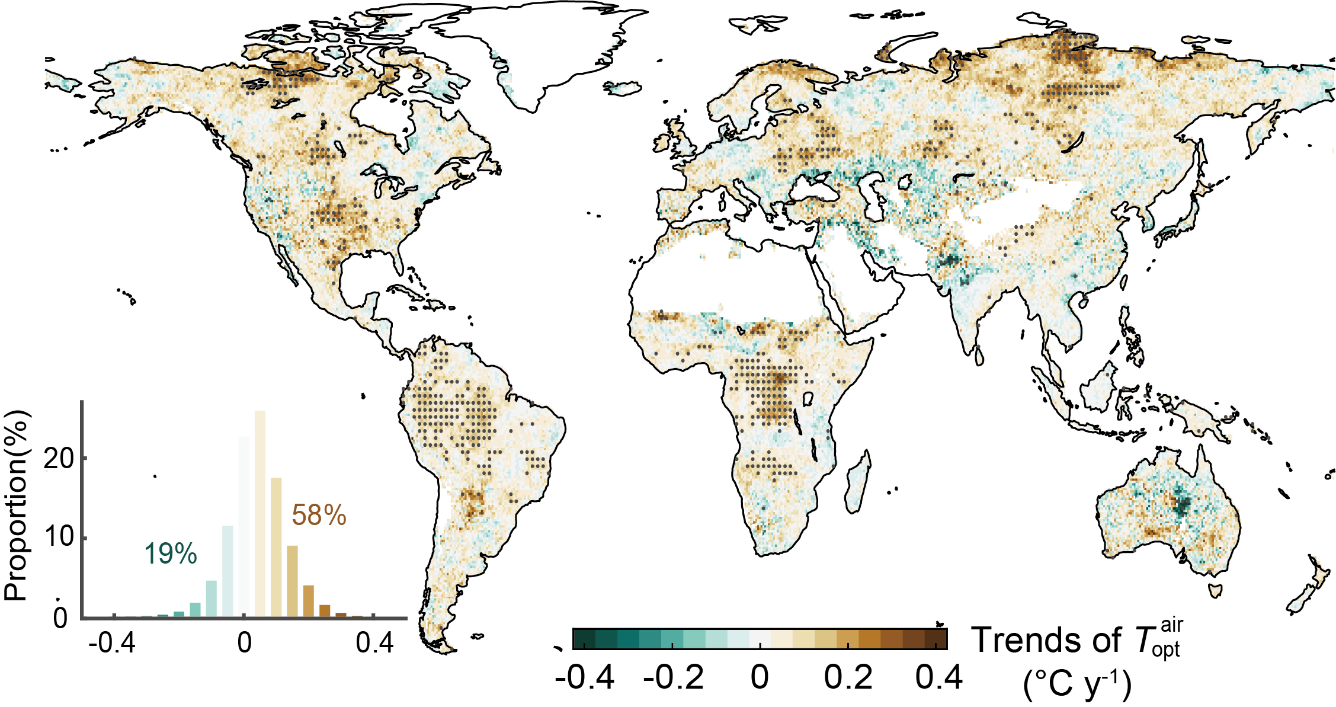


**Figure S7. Spatial pattern of the trend in *Tair opt* for the period 2003-2024.** Dot signs(·) denote trends significant at the 0.05 level (*P*<0.05). The inset histogram represents the areal proportion for different trends in magnitude and the total percentage of areas as annotated.

**
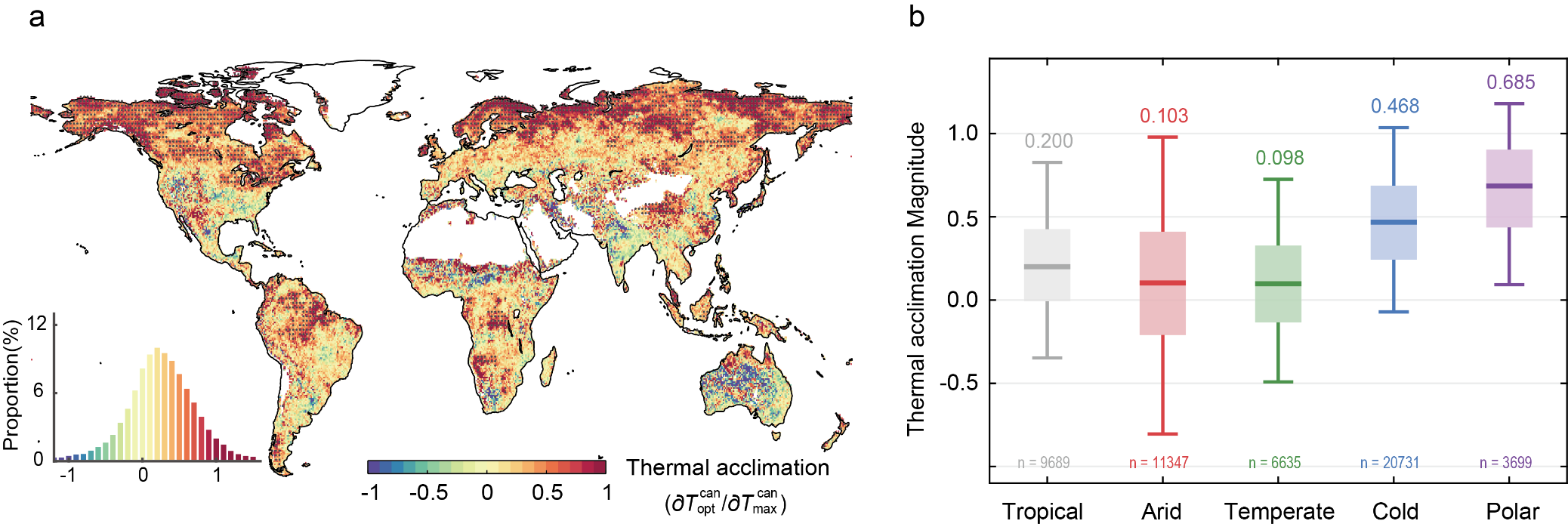
**

**Figure S8. The magnitude of thermal acclimation over the last two decades, derived from air temperature. a,** Spatial distribution of the magnitude of thermal acclimation, defined as the ratio ∂*T*air opt/∂*T*air max over the last 22 years at each grid point. Regions with significant ratios (*P*<0.05) were marked with dot symbols. The inset histogram shows the distribution of acclimation magnitudes. **b,** difference in thermal acclimation magnitude across major climate zones. Horizontal lines denote medians, boxes represent interquartile ranges, and whiskers span the 5^th^–95^th^ percentiles. Median values are displayed above each box, and sample sizes (n) are shown below. Climate zones were defined according to the 1991–2020 Köppen–Geiger climate classification.

**
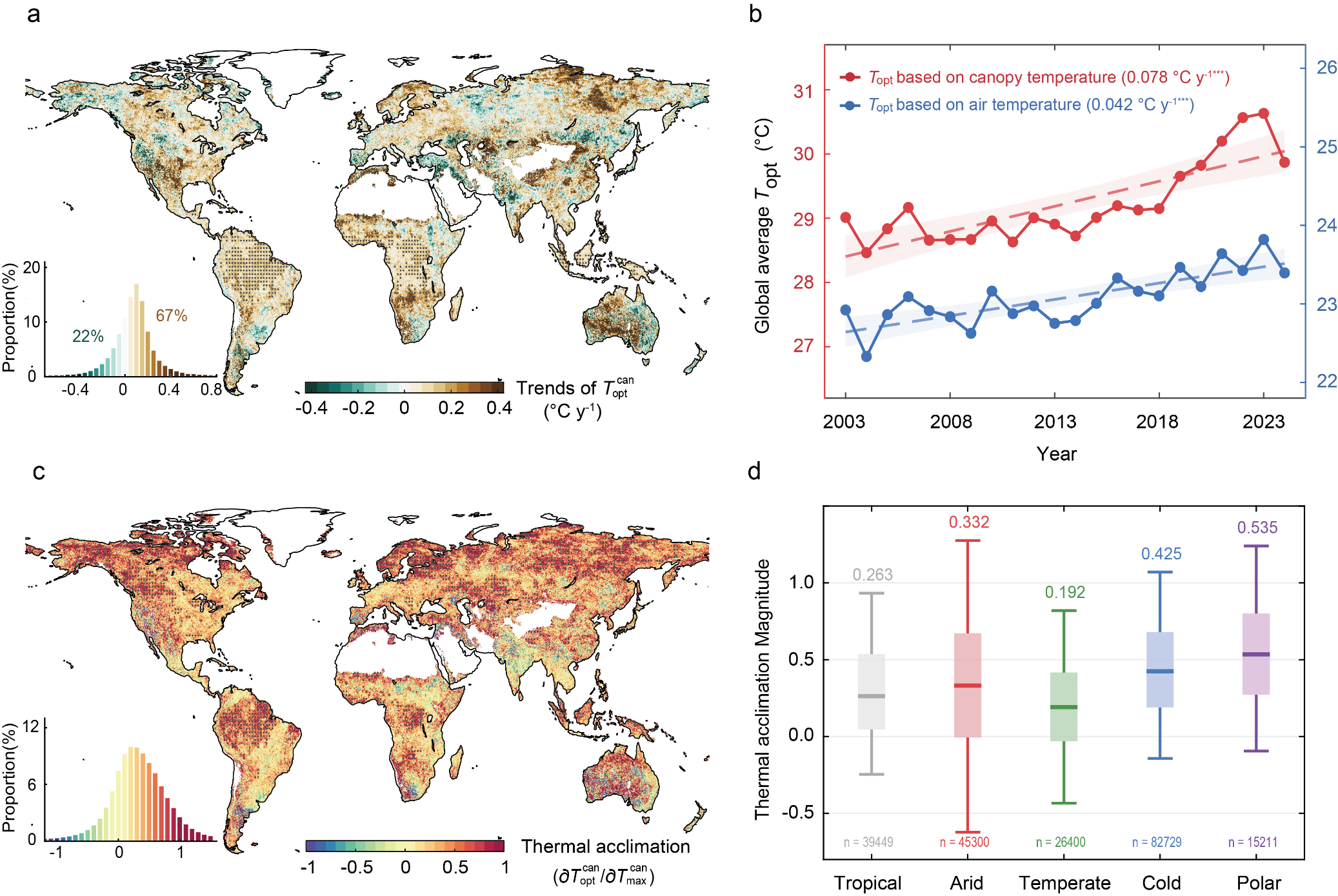
**

**Figure S9. Temporal changes of *T*can opt and its magnitude of thermal acclimation over the last two decades, based on the NIR_V_ proxy. a,** Spatial pattern of trends in *T*can opt from 2003 to 2024. Dot markers(·) denote grid regions where the trend is significant at the 0.05 level (P<0.05). The inset histogram shows the areal proportion across different trend magnitudes, with the total percentage annotated. **b,** The temporal changes of globally averaged *T*can opt and *T*air opt for the period 2003–2024. The solid line shows annual global means, and the dashed line represents the trend fitted using least squares regression and its 95% confidence interval as a grey plume. ^***^, *P*<0.001. **c,** Spatial distribution of the magnitude of thermal acclimation, defined as the ratio ∂*T*can opt/∂*T*can max over the last 22 years at each grid point. Regions with significant ratios (*P*<0.05) were marked with dot symbols. The inset histogram shows the distribution of acclimation magnitudes. **d,** difference in thermal acclimation magnitude across major climate zones. Horizontal lines denote medians, boxes represent interquartile ranges, and whiskers span the 5^th^–95^th^ percentiles. Median values are displayed above each box, and sample sizes (n) are shown below. Climate zones were defined according to the 1991–2020 Köppen–Geiger climate classification.

**
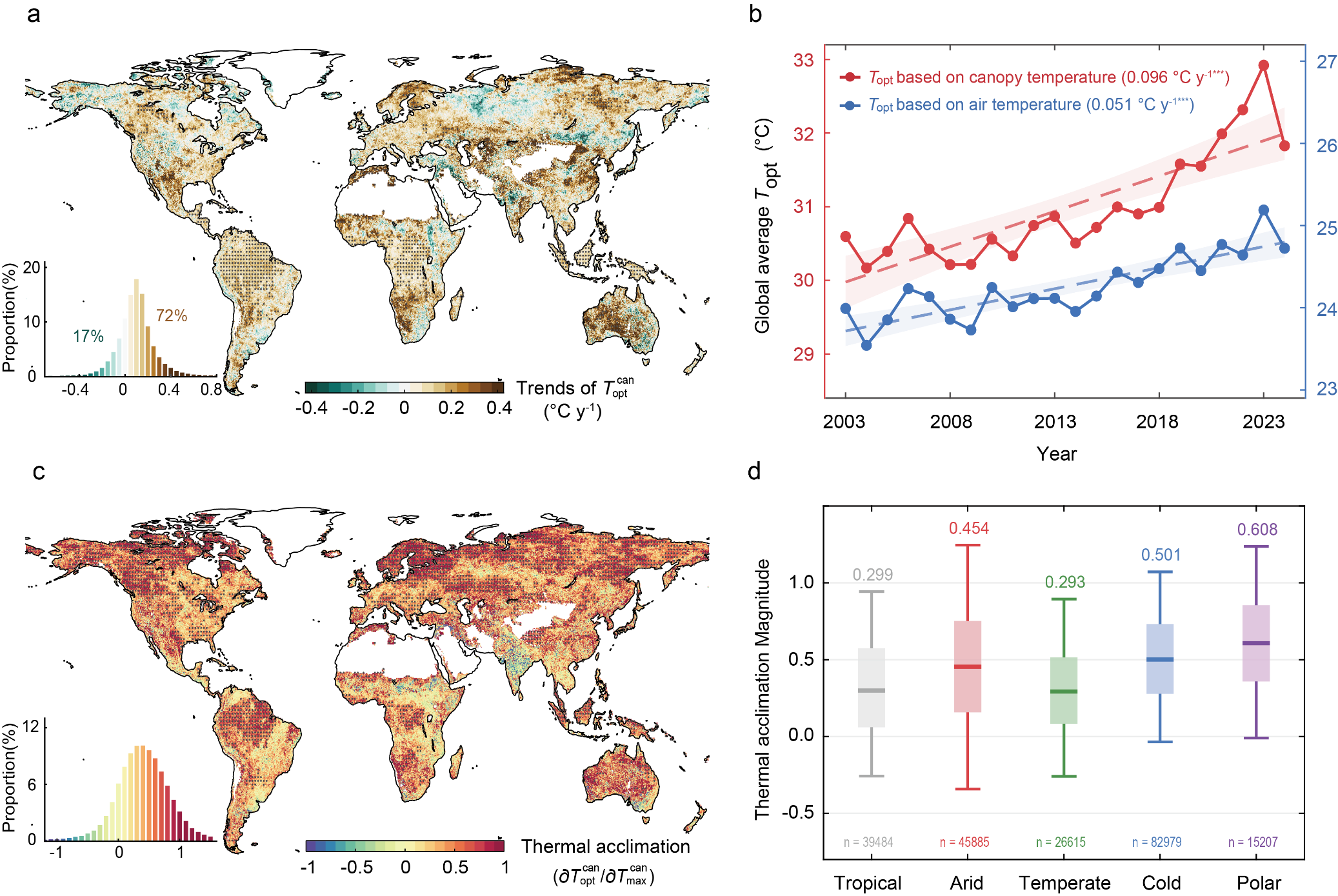
**

**Figure S10. Temporal changes of *T*can opt and its magnitude of thermal acclimation over the last two decades, based on the NIR_V_P proxy. a,** Spatial pattern of trends in *T*can opt from 2003 to 2024. Dot markers(·) denote grid regions where the trend is significant at the 0.05 level (P<0.05). The inset histogram shows the areal proportion across different trend magnitudes, with the total percentage annotated. **b,** The temporal changes of globally averaged *T*can opt and *T*air opt for the period 2003–2024. The solid line shows annual global means, and the dashed line represents the trend fitted using least squares regression and its 95% confidence interval as a grey plume. ^***^, *P*<0.001. **c,** Spatial distribution of the magnitude of thermal acclimation, defined as the ratio ∂*T*can opt/∂*T*can max over the last 22 years at each grid point. Regions with significant ratios (*P*<0.05) were marked with dot symbols. The inset histogram shows the distribution of acclimation magnitudes. **d,** difference in thermal acclimation magnitude across major climate zones. Horizontal lines denote medians, boxes represent interquartile ranges, and whiskers span the 5^th^–95^th^ percentiles. Median values are displayed above each box, and sample sizes (n) are shown below. Climate zones were defined according to the 1991–2020 Köppen–Geiger climate classification.

**
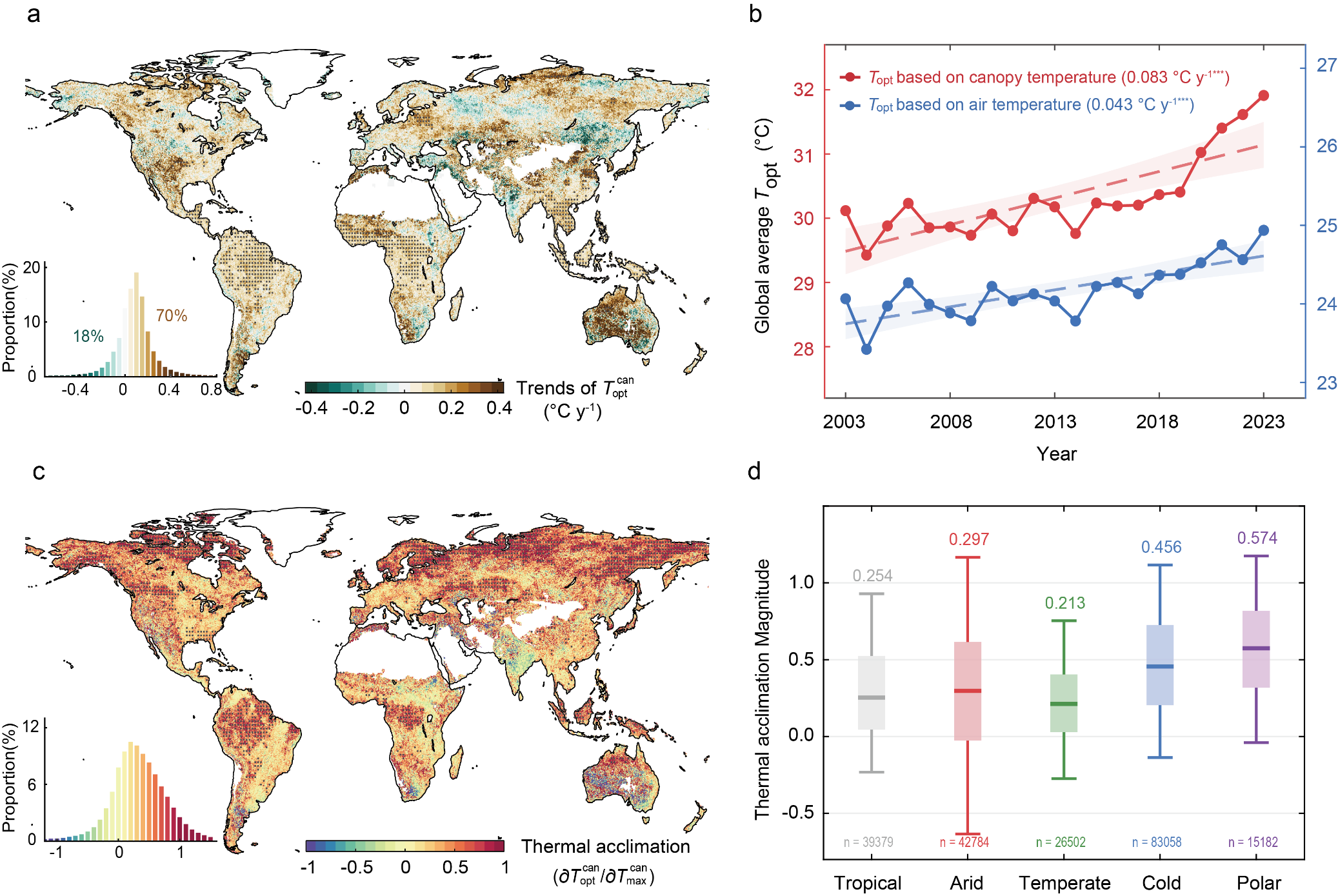
**

**Figure S11. Temporal changes of *T*can opt and its magnitude of thermal acclimation over the last two decades, based on the CSIF proxy. a,** Spatial pattern of trends in *T*can opt from 2003 to 2024. Dot markers(·) denote grid regions where the trend is significant at the 0.05 level (P<0.05). The inset histogram shows the areal proportion across different trend magnitudes, with the total percentage annotated. **b,** The temporal changes of globally averaged *T*can opt and *T*air opt for the period 2003–2024. The solid line shows annual global means, and the dashed line represents the trend fitted using least squares regression and its 95% confidence interval as a grey plume. ^***^, *P*<0.001. **c,** Spatial distribution of the magnitude of thermal acclimation, defined as the ratio ∂*T*can opt/∂*T*can max over the last 22 years at each grid point. Regions with significant ratios (*P*<0.05) were marked with dot symbols. The inset histogram shows the distribution of acclimation magnitudes. **d,** difference in thermal acclimation magnitude across major climate zones. Horizontal lines denote medians, boxes represent interquartile ranges, and whiskers span the 5^th^–95^th^ percentiles. Median values are displayed above each box, and sample sizes (n) are shown below. Climate zones were defined according to the 1991–2020 Köppen–Geiger climate classification.


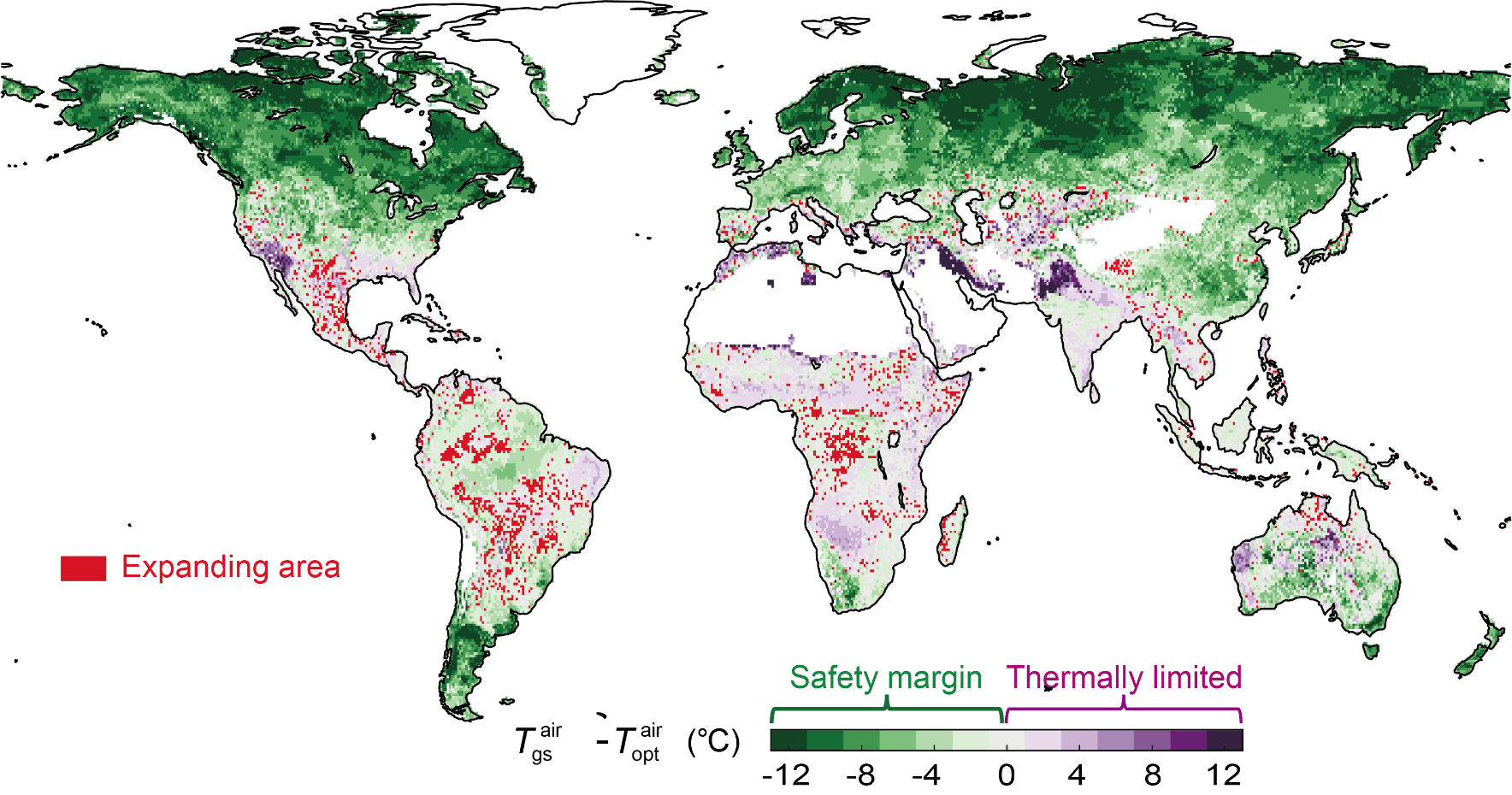


**Figure S12. Patterns of the difference between *T*air opt and *T*air gs.** The difference between the optimal air temperature (*T*air opt) (and so calculated similarly to the *T*air opt values in Fig. S1, and not including acclimate) and the multiyear mean growing-season average daily maximum air temperature (*T*air gs) during the period 2003–2024 inclusive. When *T*air opt is higher than *T*air gs, the difference represents a safety margin for vegetation productivity under warming; conversely, when *T*air gs exceeds *T*air opt, the area is already subject to thermal stress, with the difference illustrating the extent of thermal limitations on photosynthesis. This stress is due to the asymmetry of the photosynthetic response, where even a small increment in temperature above *T*air opt results in a disproportionately large suppression of productivity. The expansion of thermally limited areas without acclimation is depicted as a red overlay, calculated as present where *T*air gs was lower than *T*air opt during the first five years (2003–2007) but exceeded *T*air opt in the last five years (2020–2024).

**
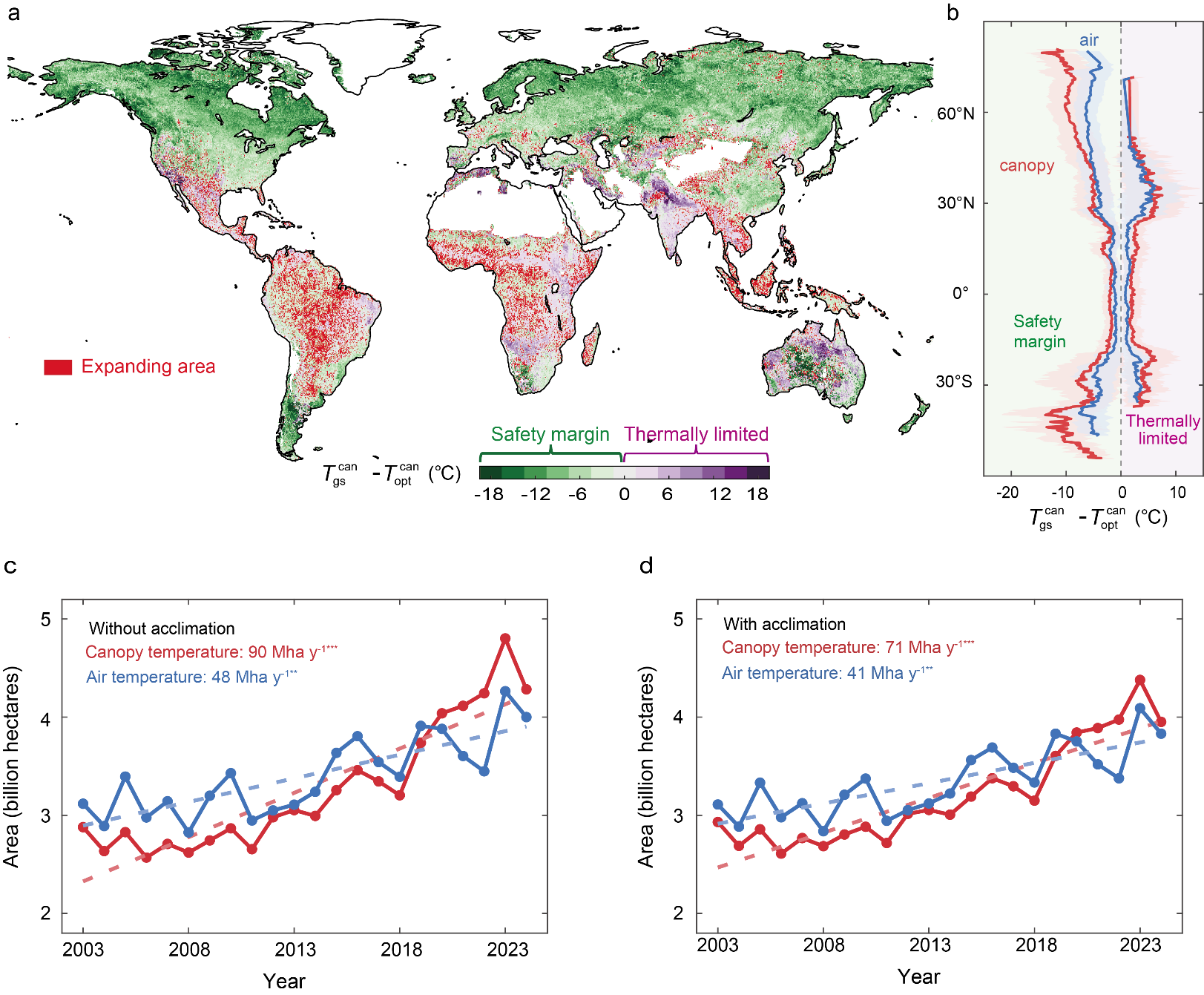
**

**Figure S13. Patterns of the difference between** ***T*can opt and *T*can gs, and the expansion of thermally limited areas, based on the NIR_V_ proxy. a,** The difference between the optimal canopy temperature (*T*can opt) (and so calculated similarly to the *T*can opt values in Fig. 1, and not including acclimate) and the multiyear mean growing-season average daily maximum canopy temperature (*T*can gs) during the period 2003–2024 inclusive. When *T*can opt is higher than *T*can gs, the difference represents a safety margin for vegetation productivity under warming; conversely, when *T*can gs exceeds *T*can opt, the area is already subject to thermal stress, with the difference illustrating the extent of thermal limitations on photosynthesis. This stress is due to the asymmetry of the photosynthetic response, where even a small increment in temperature above *T*can opt results in a disproportionately large suppression of productivity. The expansion of thermally limited areas without acclimation is depicted in panel **a** as a red overlay, calculated as present where *T*can gs was lower than *T*can opt during the first five years (2003–2007) but exceeded *T*can opt in the last five years (2020–2024), corresponding to the red line in panel **c**. **b**, The latitudinal average of *T*can opt - *T*can gs based on canopy and air temperatures. Regions with safety margins and thermal limitations are calculated separately to prevent the cancellation of positive and negative values. The combined distributional pattern of *T*air opt-*T*air gs is shown in Fig. S12. **c, d,** Yearly changes over the last 22 years in the total area of thermally limited regions where the mean growing-season temperature exceeded its optimal value (i.e., where *T*can gs>*T*can opt). Panel **c** illustrates changes without considering thermal acclimation, and panel **d** incorporates acclimation. In panels **c** and **d**, the dashed lines represent linear-trend fits. ^**^, *P*<0.01; ^***^, *P*<0.001.

**
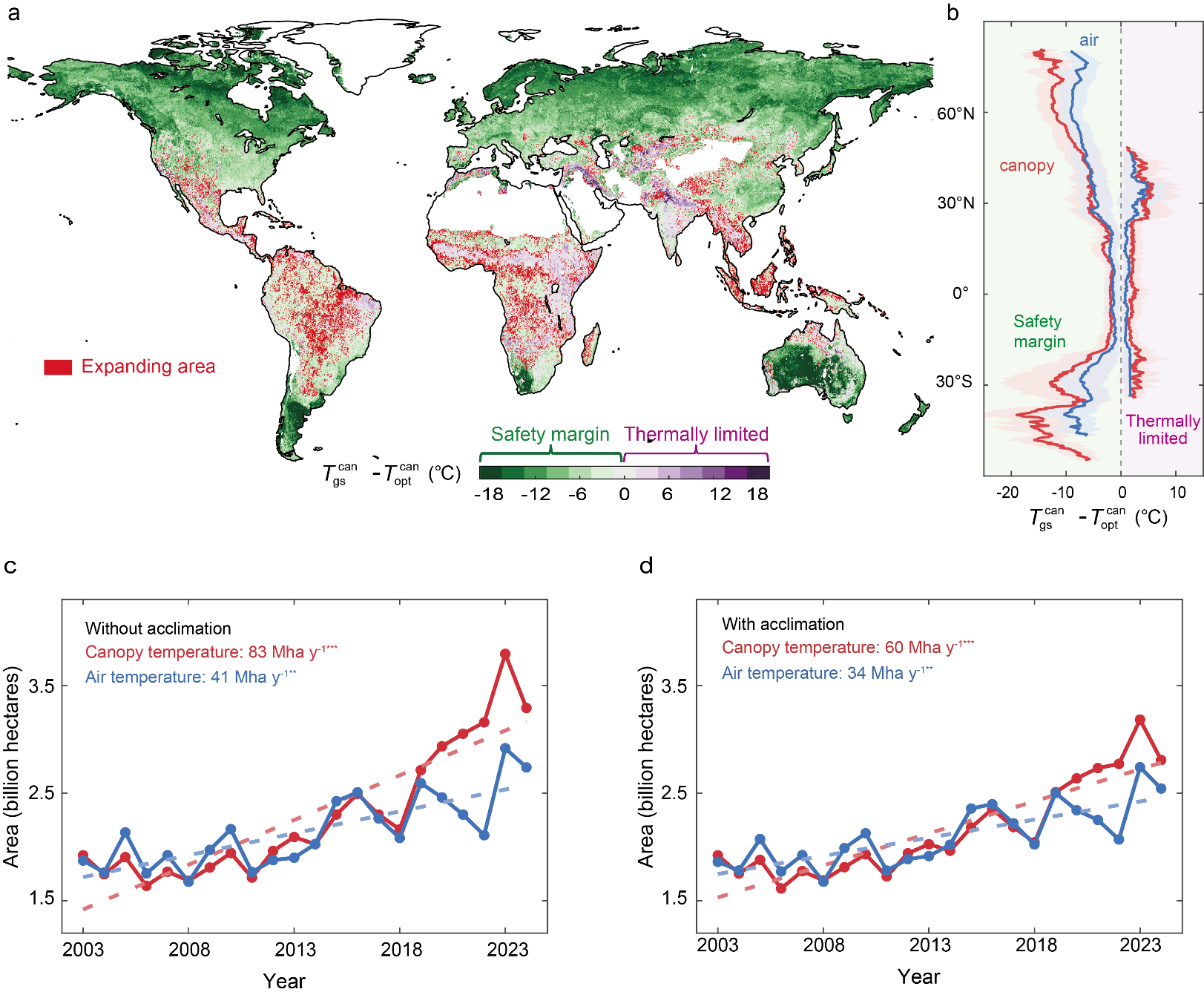
**

**Figure S14. Patterns of the difference between *T*can opt and *T*can gs, and the expansion of thermally limited areas, based on the NIR_V_P proxy. a,** The difference between the optimal canopy temperature (*T*can opt) (and so calculated similarly to the *T*can opt values in Fig. 1, and not including acclimate) and the multiyear mean growing-season average daily maximum canopy temperature (*T*can gs) during the period 2003–2024 inclusive. When *T*can opt is higher than *T*can gs, the difference represents a safety margin for vegetation productivity under warming; conversely, when *T*can gs exceeds *T*can opt, the area is already subject to thermal stress, with the difference illustrating the extent of thermal limitations on photosynthesis. This stress is due to the asymmetry of the photosynthetic response, where even a small increment in temperature above *T*can opt results in a disproportionately large suppression of productivity. The expansion of thermally limited areas without acclimation is depicted in panel **a** as a red overlay, calculated as present where *T*can gs was lower than *T*can opt during the first five years (2003–2007) but exceeded *T*can opt in the last five years (2020–2024), corresponding to the red line in panel **c**. **b**, The latitudinal average of *T*can opt - *T*can gs based on canopy and air temperatures. Regions with safety margins and thermal limitations are calculated separately to prevent the cancellation of positive and negative values. The combined distributional pattern of *T*air opt-*T*air gs is shown in Fig. S12. **c, d,** Yearly changes over the last 22 years in the total area of thermally limited regions where the mean growing-season temperature exceeded its optimal value (i.e., where *T*can gs>*T*can opt). Panel **c** illustrates changes without considering thermal acclimation, and panel **d** incorporates acclimation. In panels **c** and **d**, the dashed lines represent linear-trend fits. ^**^, *P*<0.01; ^***^, *P*<0.001.

**
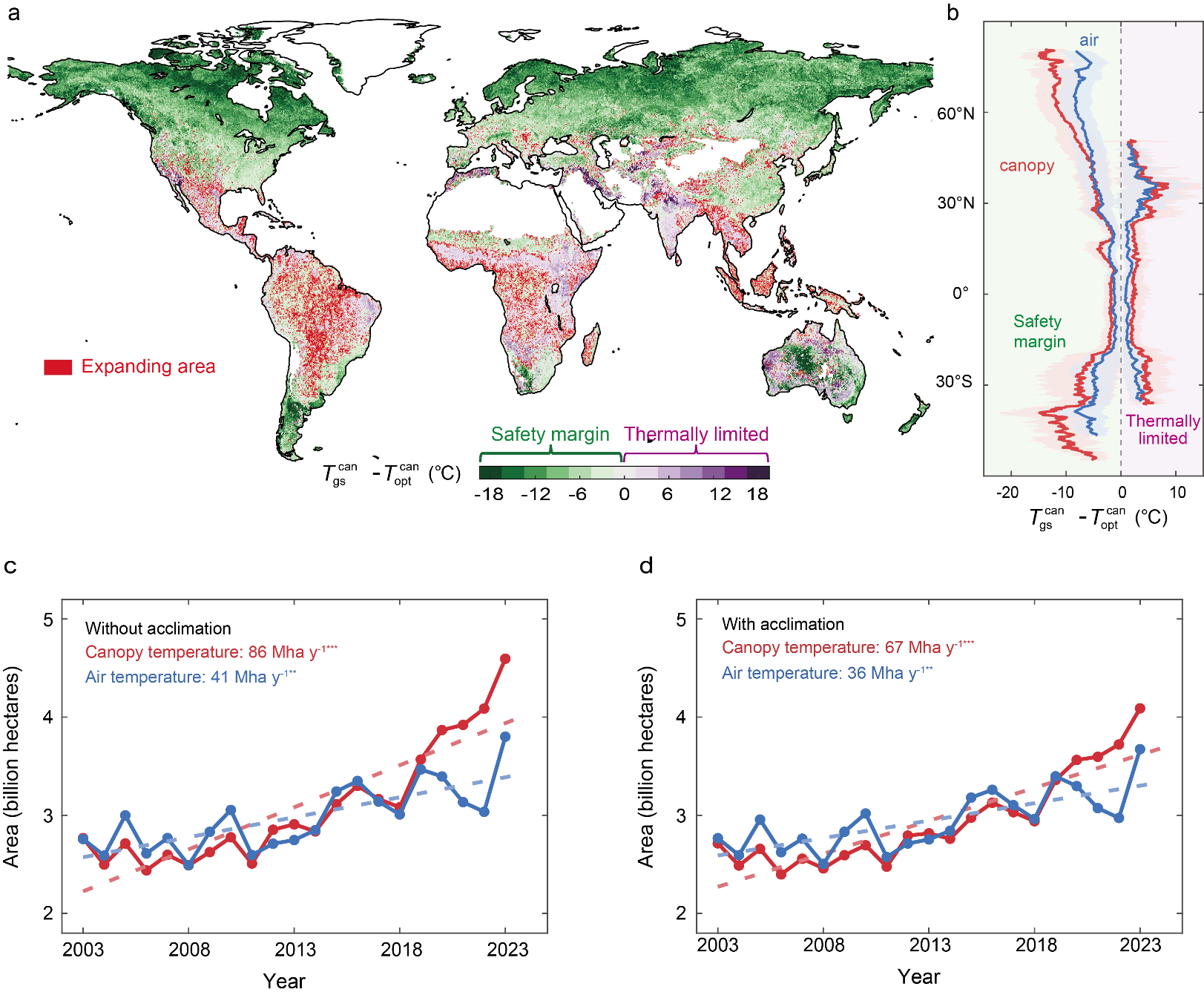
**

**Figure S15. Patterns of the difference between *T*can opt and *T*can gs, and the expansion of thermally limited areas, based on the CSIF proxy. a,** The difference between the optimal canopy temperature (*T*can opt) (and so calculated similarly to the *T*can opt values in Fig. 1, and not including acclimate) and the multiyear mean growing-season average daily maximum canopy temperature (*T*can gs) during the period 2003–2024 inclusive. When *T*can opt is higher than *T*can gs, the difference represents a safety margin for vegetation productivity under warming; conversely, when *T*can gs exceeds *T*can opt, the area is already subject to thermal stress, with the difference illustrating the extent of thermal limitations on photosynthesis. This stress is due to the asymmetry of the photosynthetic response, where even a small increment in temperature above *T*can opt results in a disproportionately large suppression of productivity. The expansion of thermally limited areas without acclimation is depicted in panel **a** as a red overlay, calculated as present where *T*can gs was lower than *T*can opt during the first five years (2003–2007) but exceeded *T*can opt in the last five years (2020–2024), corresponding to the red line in panel **c**. **b**, The latitudinal average of *T*can opt - *T*can gs based on canopy and air temperatures. Regions with safety margins and thermal limitations are calculated separately to prevent the cancellation of positive and negative values. The combined distributional pattern of *T*air opt-*T*air gs is shown in Fig. S12. **c, d,** Yearly changes over the last 22 years in the total area of thermally limited regions where the mean growing-season temperature exceeded its optimal value (i.e., where *T*can gs>*T*can opt). Panel **c** illustrates changes without considering thermal acclimation, and panel **d** incorporates acclimation. In panels **c** and **d**, the dashed lines represent linear-trend fits. ^**^, *P*<0.01; ^***^, *P*<0.001.


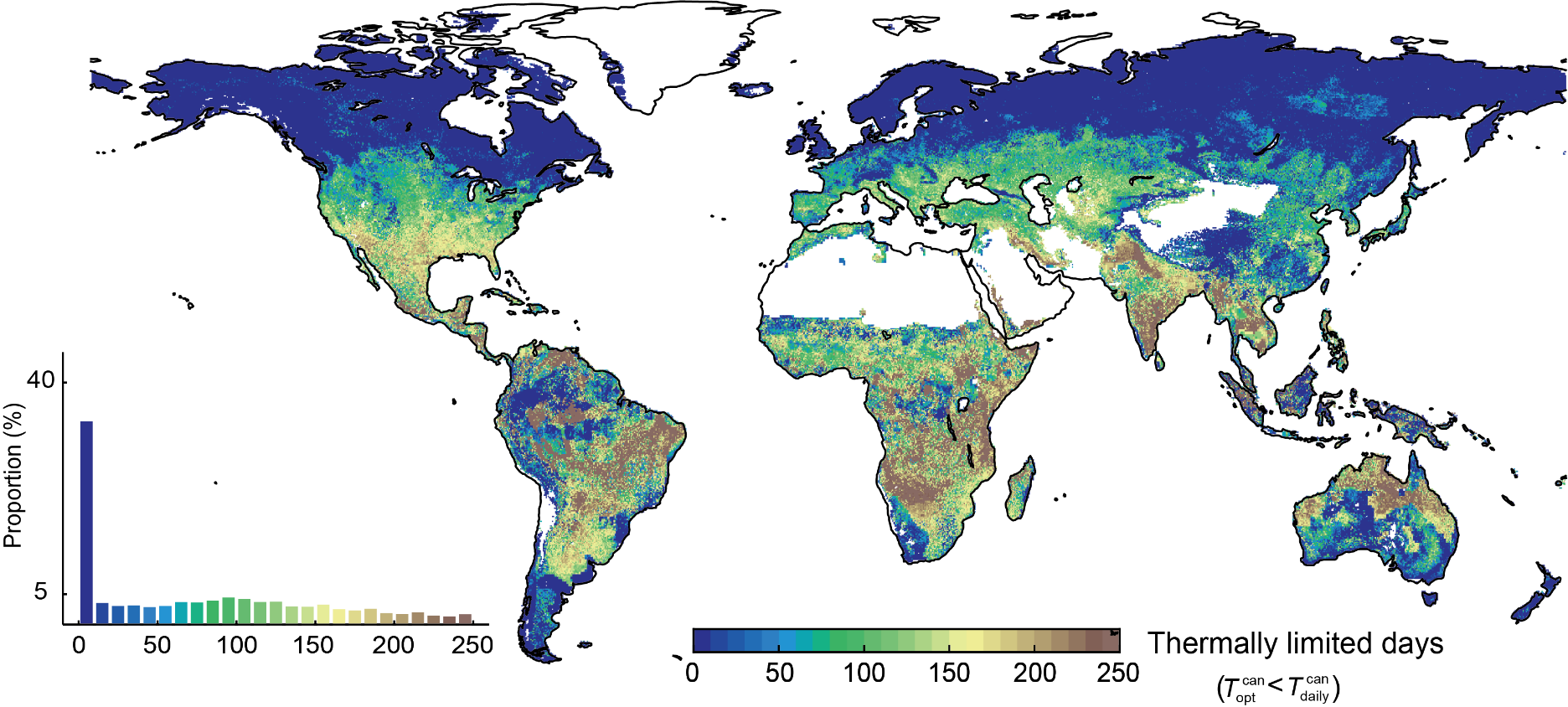


**Figure S16. Number of high-temperature limited days during 2003-2024 based on *T*can opt.** High-temperature limited days are defined as the number of days in a year when the daily maximum canopy temperature exceeds *T*can opt. The inset histogram represents the areal proportion of different numbers of days.


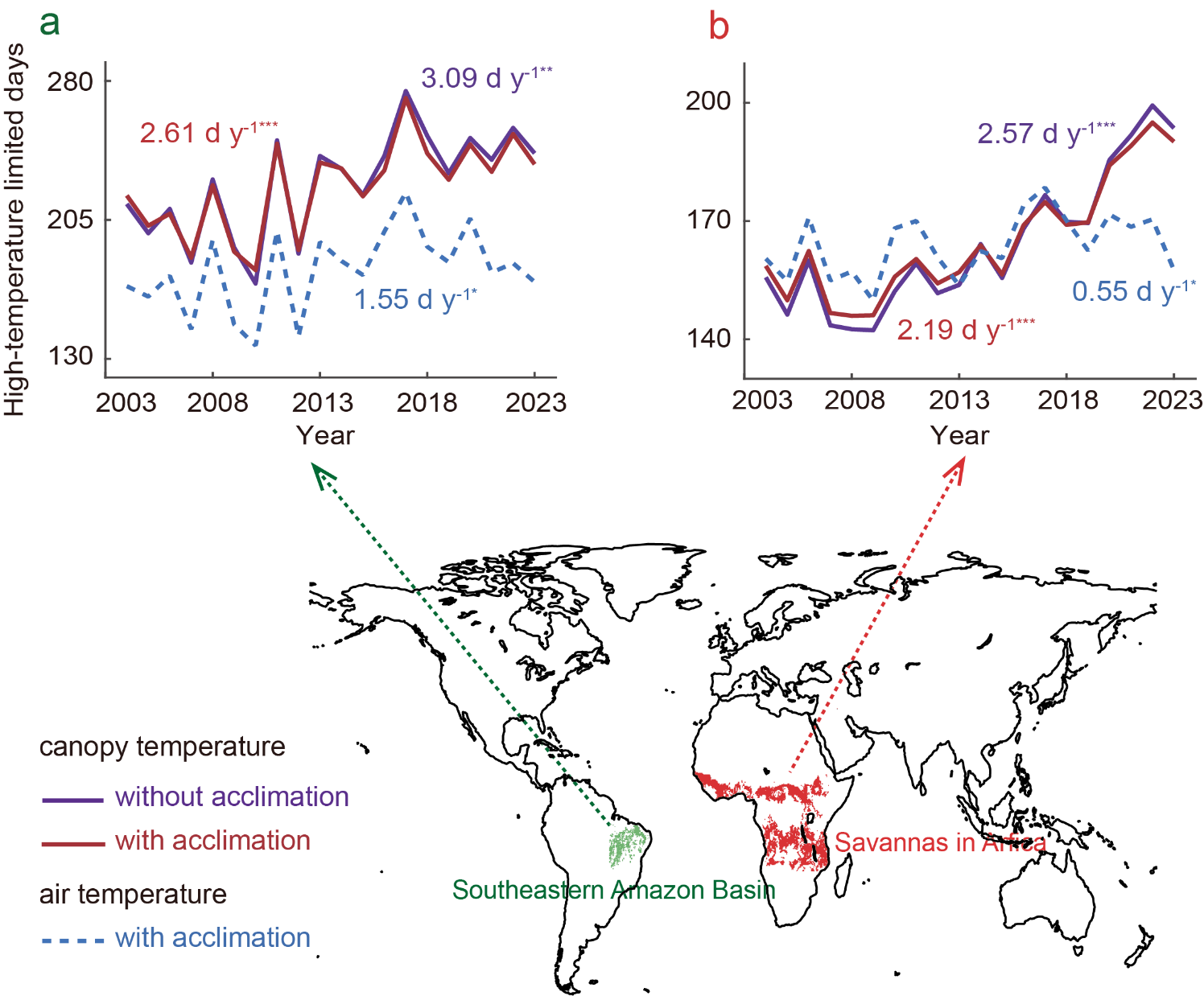


**Figure S17. Variations in the number of high-temperature days over the last 22 years for the southeastern Amazon Basin and savannas in Africa. a, b** Interannual variations and trends in the number of high-temperature limited days for the southeastern Amazon Basin and savannas in Africa. In each panel, the purple curves are calculated using canopy temperature but without allowing for acclimation in *T*can opt. The red curves are identical to the purple curves, except that acclimation is incorporated in *T*can opt. As a technical comparison, we also show calculations allowing for acclimation, but instead forced with air temperatures, *T*air opt. Statistical significance for all text annotations are: *, P<0.1; **, P<0.01; and ***, P<0.001.


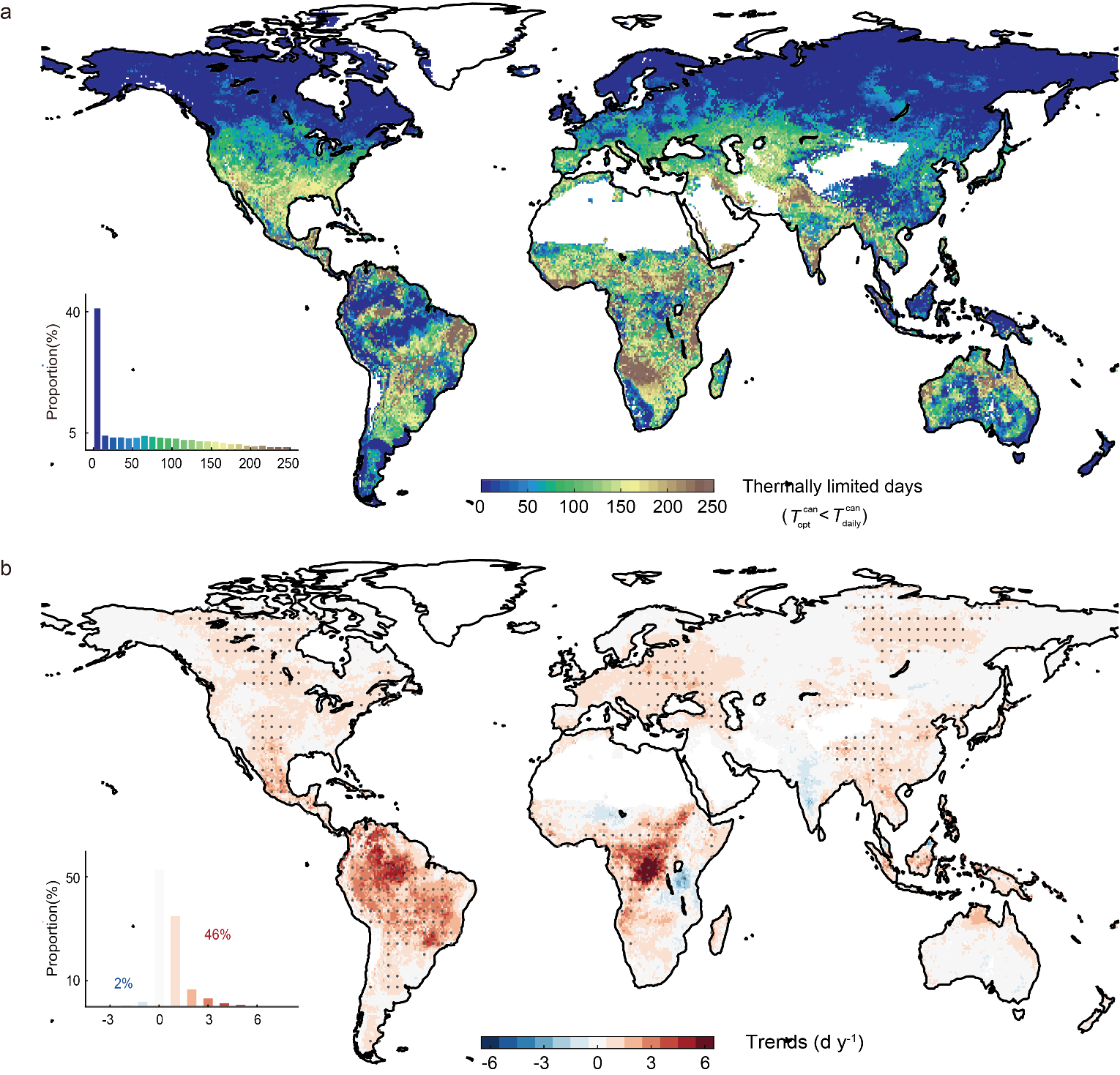


**Figure S18. Number of high-temperature limited days and its trends over the last 22 years based on *T*air opt without acclimation. a,** Distribution of high-temperature limited days for the period 2003–2024 inclusive. High-temperature limited days are defined as the number of days in a year when the daily maximum air temperature exceeds *T*air opt. The inset histogram represents the areal proportion for different day magnitudes. b, Spatial distribution of trends in the frequency of photosynthesis subjected to thermal limitation during the growing period and from years 2003 to 2024. Calculations are based on crossing derived spatially-specific values of *T*air opt, but assuming the latter are invariant in time, and thus without thermal acclimation. Trends were calculated using linear regression and the standard least squares method, and regions with significant ratios (*P*<0.05) were marked with dot symbols. The inset histogram represents the areal proportion for different trend magnitudes, binned in intervals of 1d y^-1^.


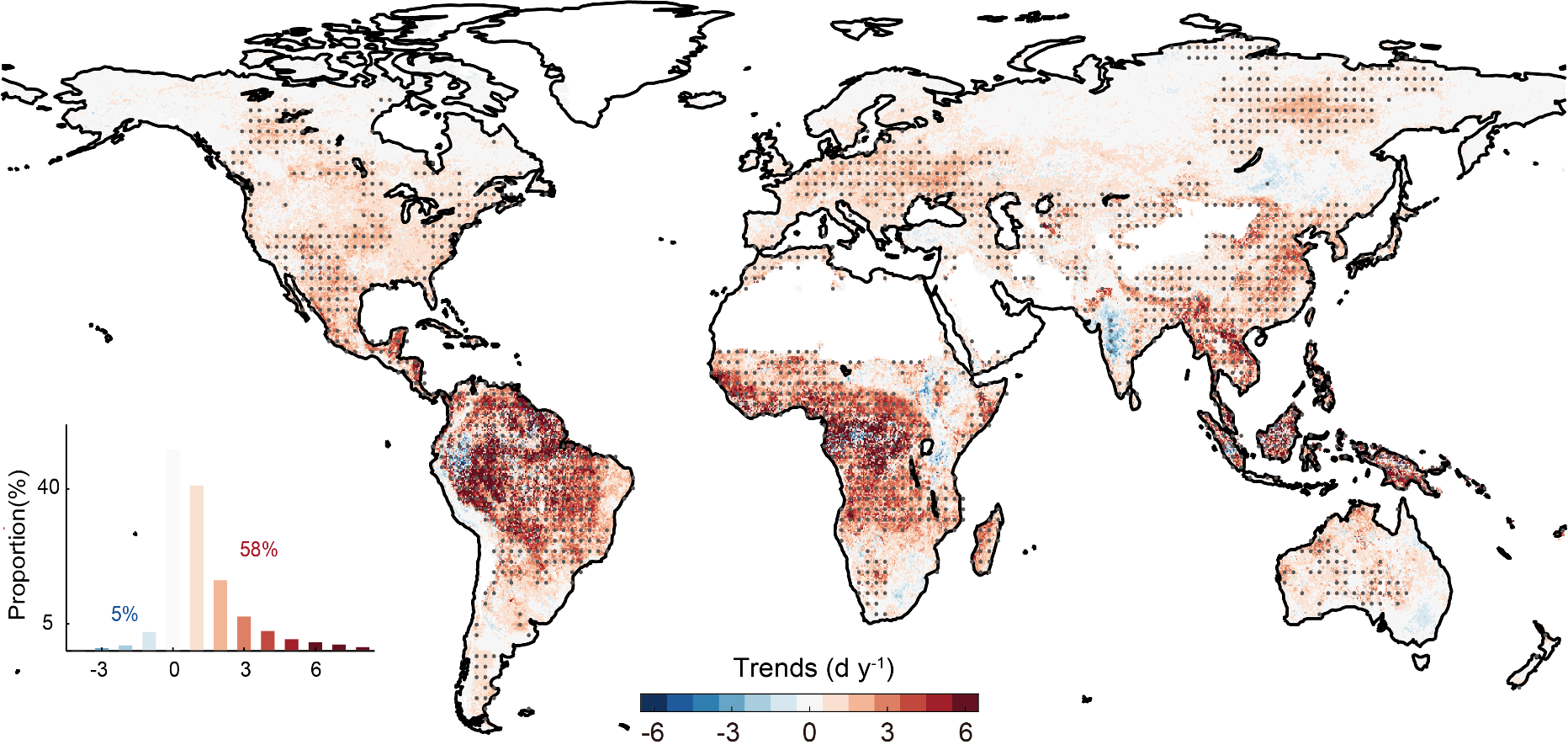


**Figure S19. Trends in the number of high-temperature limited days over the last 22 years based on *Tcan opt* with acclimation.** Spatial distribution of trends in the frequency of photosynthesis subjected to thermal limitation during the growing period and from 2003 to 2022. Calculations are based on crossing derived spatially-specific values of *T*can opt, but assuming the latter are variant in time, and thus with thermal acclimation. Trends were calculated using linear regression and the standard least squares method, and regions with significant ratios (*P*<0.05) were marked with dot symbols. The inset histogram represents the areal proportion for different trend magnitudes, binned in intervals of 1d y^-1^.


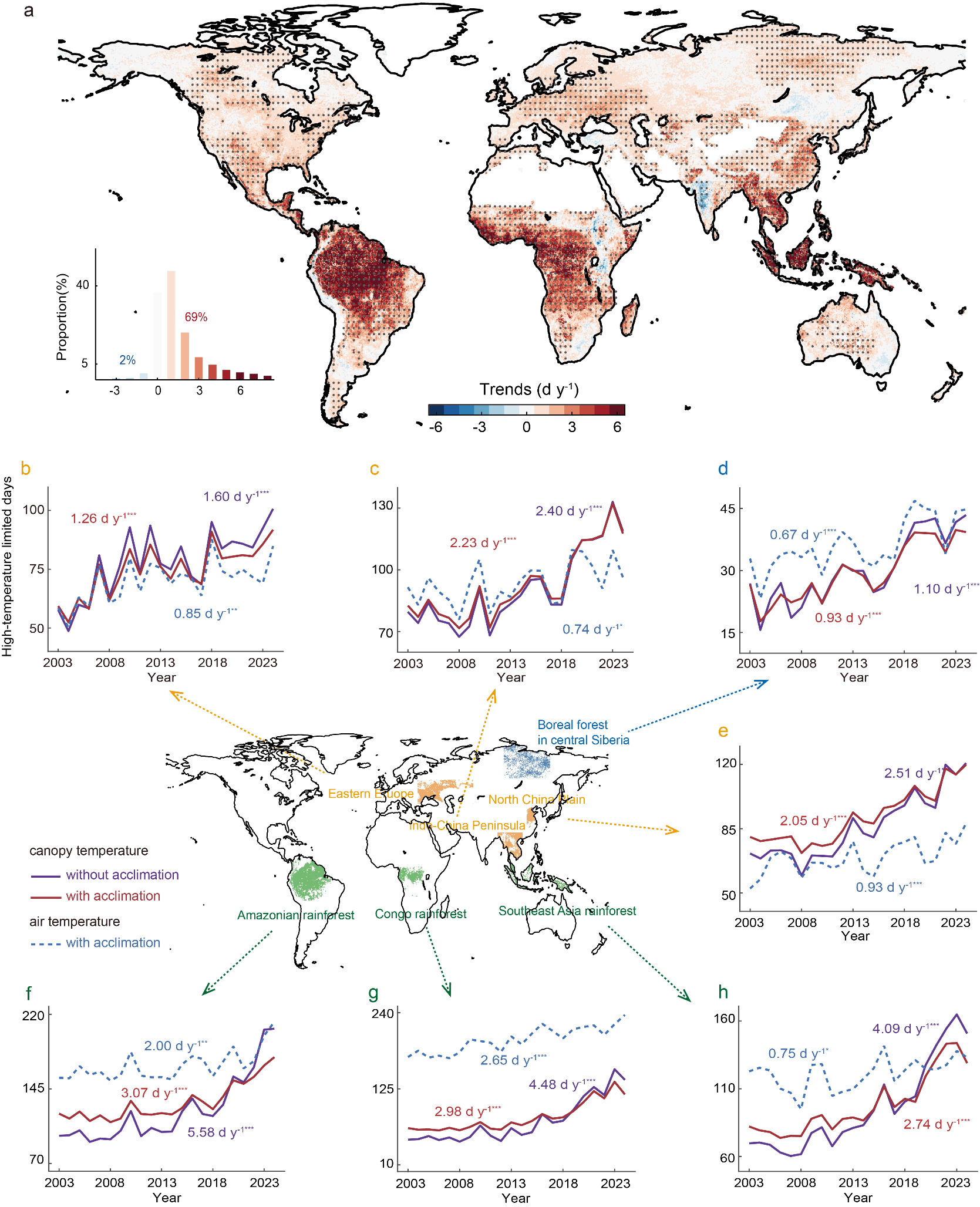


**Figure S20. Trends in the number of high-temperature days over the past 22 years, based on the NIR_V_ proxy. a,** Spatial distribution of trends in the frequency of photosynthesis subjected to thermal limitation during the growing period and from years 2003 to 2024. Calculations are based on crossing derived spatially-specific values of *T*can opt, but assuming the latter are invariant in time, and thus without thermal acclimation. Trends were calculated using linear regression and the standard least squares method, and regions with significant ratios (*P*<0.05) were marked with dot symbols. The inset histogram represents the areal proportion for different trend magnitudes, binned in intervals of 1d y^-1^. **b**–**h,** Interannual variations and trends in the number of high-temperature limited days for seven key regions as marked on the map: three agricultural regions (orange), including eastern Europe, Indo-China Peninsula and the North China Plain, boreal forests (blue) in central Siberia, three tropical rainforest regions (green) including the Amazon basin, Congo and Southeast Asia. In each panel, the purple curves are calculated using canopy temperature but without allowing for acclimation in *T*can opt. The red curves are identical to the purple curves, except that acclimation is incorporated in *T*can opt. As a technical comparison, we also show calculations allowing for acclimation, but instead forced with air temperatures, *T*air opt. Statistical significance for all text annotations are: ^*^, *P*<0.1; ^**^, *P*<0.01; and ^***^, *P*<0.001.


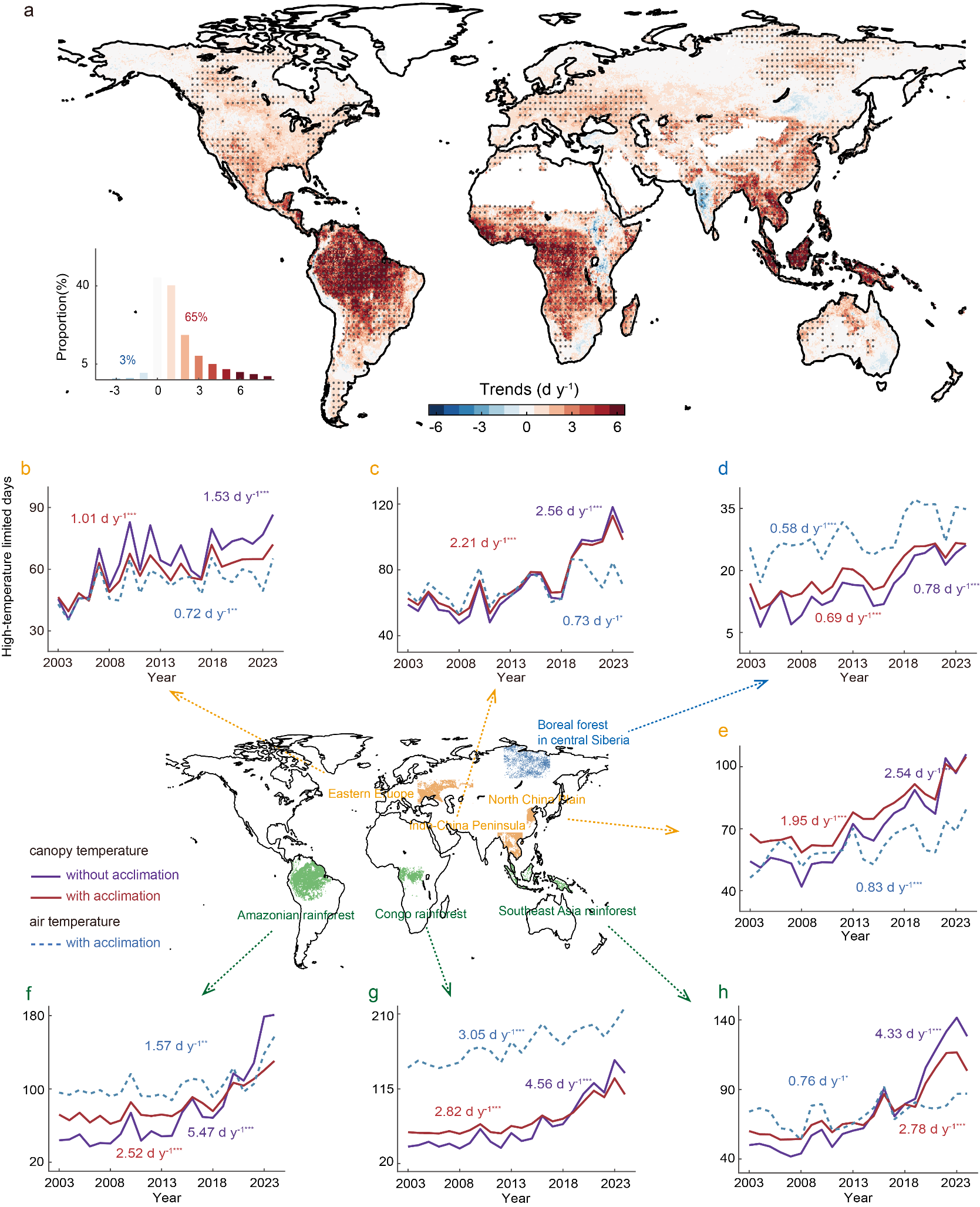


**Figure S21. Trends in the number of high-temperature days over the past 22 years, based on the NIR_V_P proxy. a,** Spatial distribution of trends in the frequency of photosynthesis subjected to thermal limitation during the growing period and from years 2003 to 2024. Calculations are based on crossing derived spatially-specific values of *T*can opt, but assuming the latter are invariant in time, and thus without thermal acclimation. Trends were calculated using linear regression and the standard least squares method, and regions with significant ratios (*P*<0.05) were marked with dot symbols. The inset histogram represents the areal proportion for different trend magnitudes, binned in intervals of 1d y^-1^. **b**–**h,** Interannual variations and trends in the number of high-temperature limited days for seven key regions as marked on the map: three agricultural regions (orange), including eastern Europe, Indo-China Peninsula and the North China Plain, boreal forests (blue) in central Siberia, three tropical rainforest regions (green) including the Amazon basin, Congo and Southeast Asia. In each panel, the purple curves are calculated using canopy temperature but without allowing for acclimation in *T*can opt. The red curves are identical to the purple curves, except that acclimation is incorporated in *T*can opt. As a technical comparison, we also show calculations allowing for acclimation, but instead forced with air temperatures, *T*air opt. Statistical significance for all text annotations are: ^*^, *P*<0.1; ^**^, *P*<0.01; and ^***^, *P*<0.001.


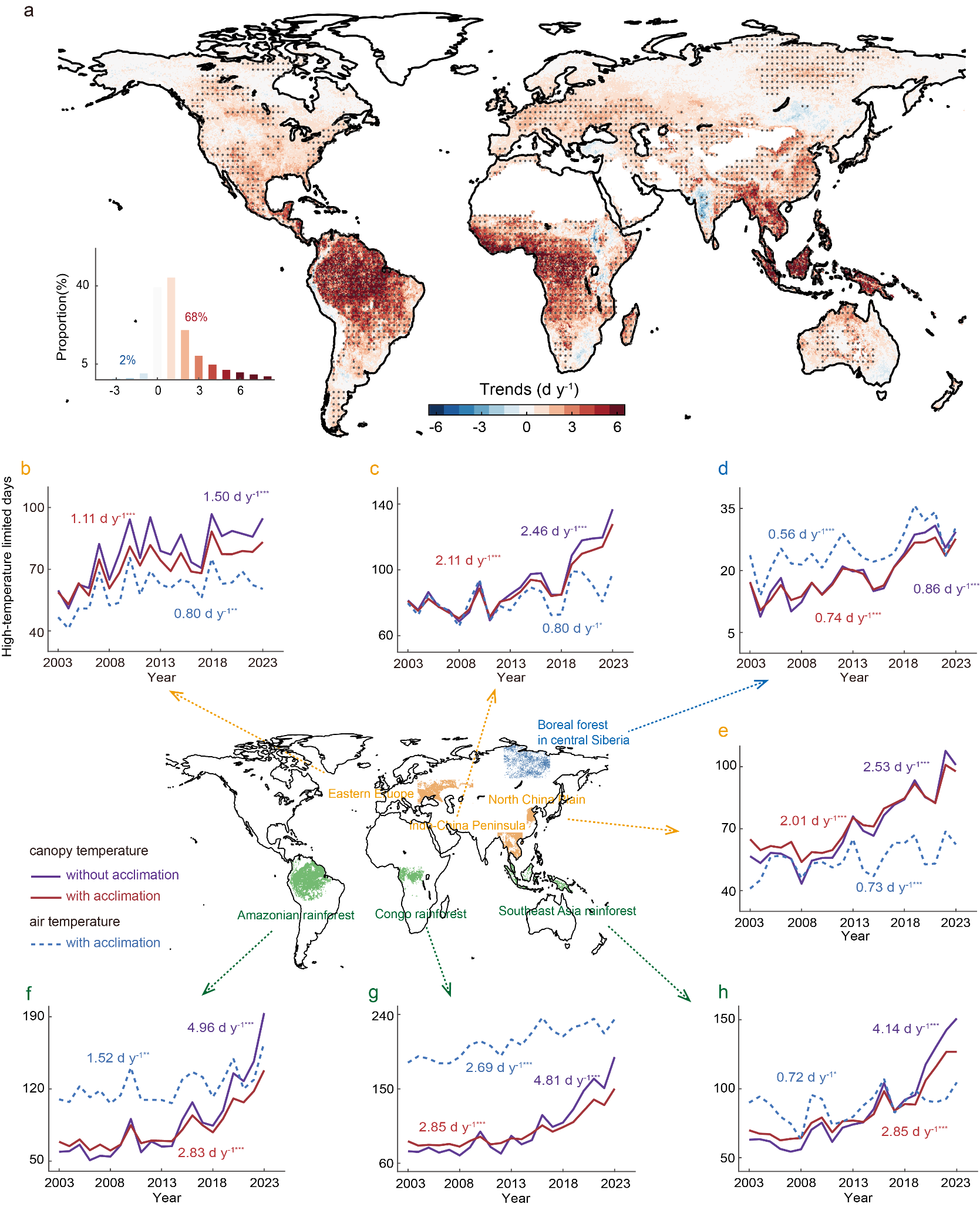


**Figure S22. Trends in the number of high-temperature days over the past 22 years, based on the CSIF proxy. a,** Spatial distribution of trends in the frequency of photosynthesis subjected to thermal limitation during the growing period and from years 2003 to 2024. Calculations are based on crossing derived spatially-specific values of *T*can opt, but assuming the latter are invariant in time, and thus without thermal acclimation. Trends were calculated using linear regression and the standard least squares method, and regions with significant ratios (*P*<0.05) were marked with dot symbols. The inset histogram represents the areal proportion for different trend magnitudes, binned in intervals of 1d y^-1^. **b**–**h,** Interannual variations and trends in the number of high-temperature limited days for seven key regions as marked on the map: three agricultural regions (orange), including eastern Europe, Indo-China Peninsula and the North China Plain, boreal forests (blue) in central Siberia, three tropical rainforest regions (green) including the Amazon basin, Congo and Southeast Asia. In each panel, the purple curves are calculated using canopy temperature but without allowing for acclimation in *T*can opt. The red curves are identical to the purple curves, except that acclimation is incorporated in *T*can opt. As a technical comparison, we also show calculations allowing for acclimation, but instead forced with air temperatures, *T*air opt. Statistical significance for all text annotations are: ^*^, *P*<0.1; ^**^, *P*<0.01; and ^***^, *P*<0.001.


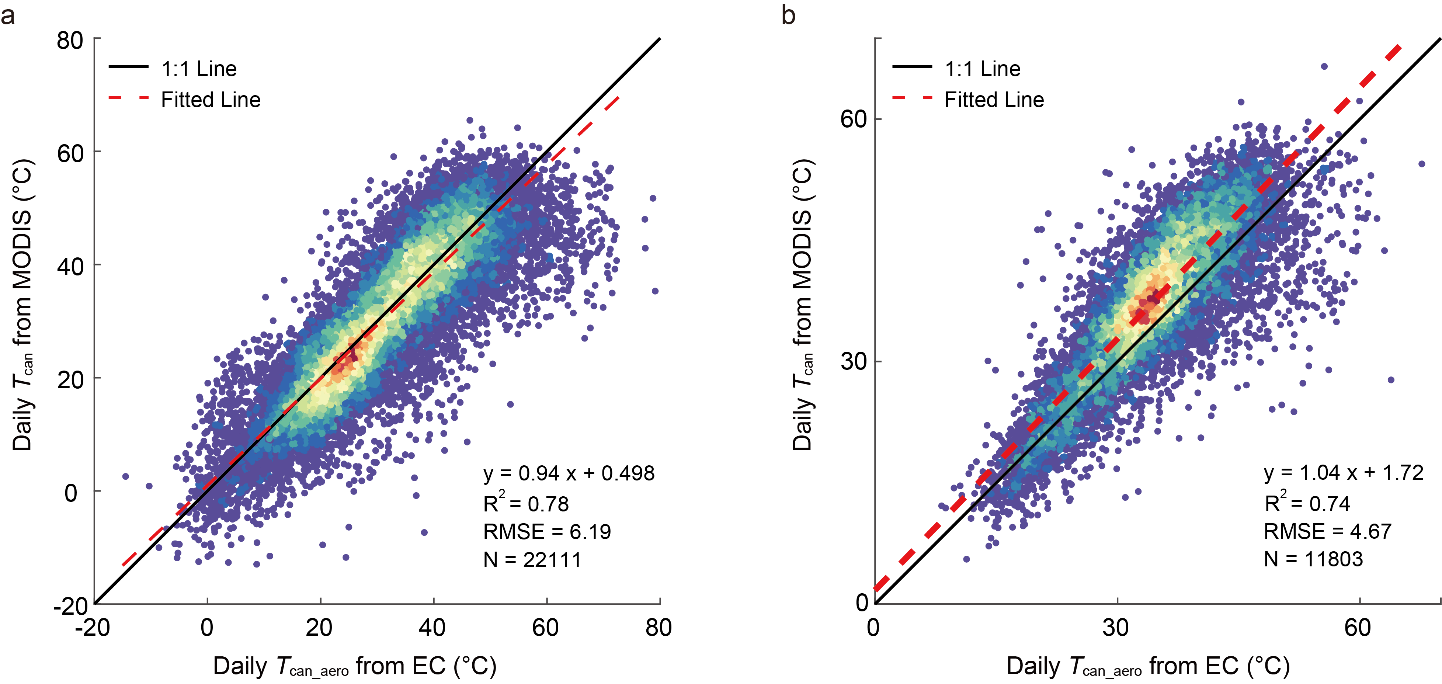


**Figure S23. The comparison between satellite-derived and eddy covariance (EC)- canopy temperature for sparsely vegetated ecosystems. a,** density scatterplots comparing satellite-derived *T*_can_ (“*y*”-axis) and EC-based *T*_can_aero_ (“*x*”-axis) for grassland ecosystems. Each point represents a paired observation during the growing season, pooled across all valid sites. The solid black line denotes the 1:1 relationship, while the red dashed line indicates the linear regression fit. Statistical metrics are reported within each panel. Warmer colors indicate higher point density. **b**, similar to **a**, but for savanna ecosystems.

**
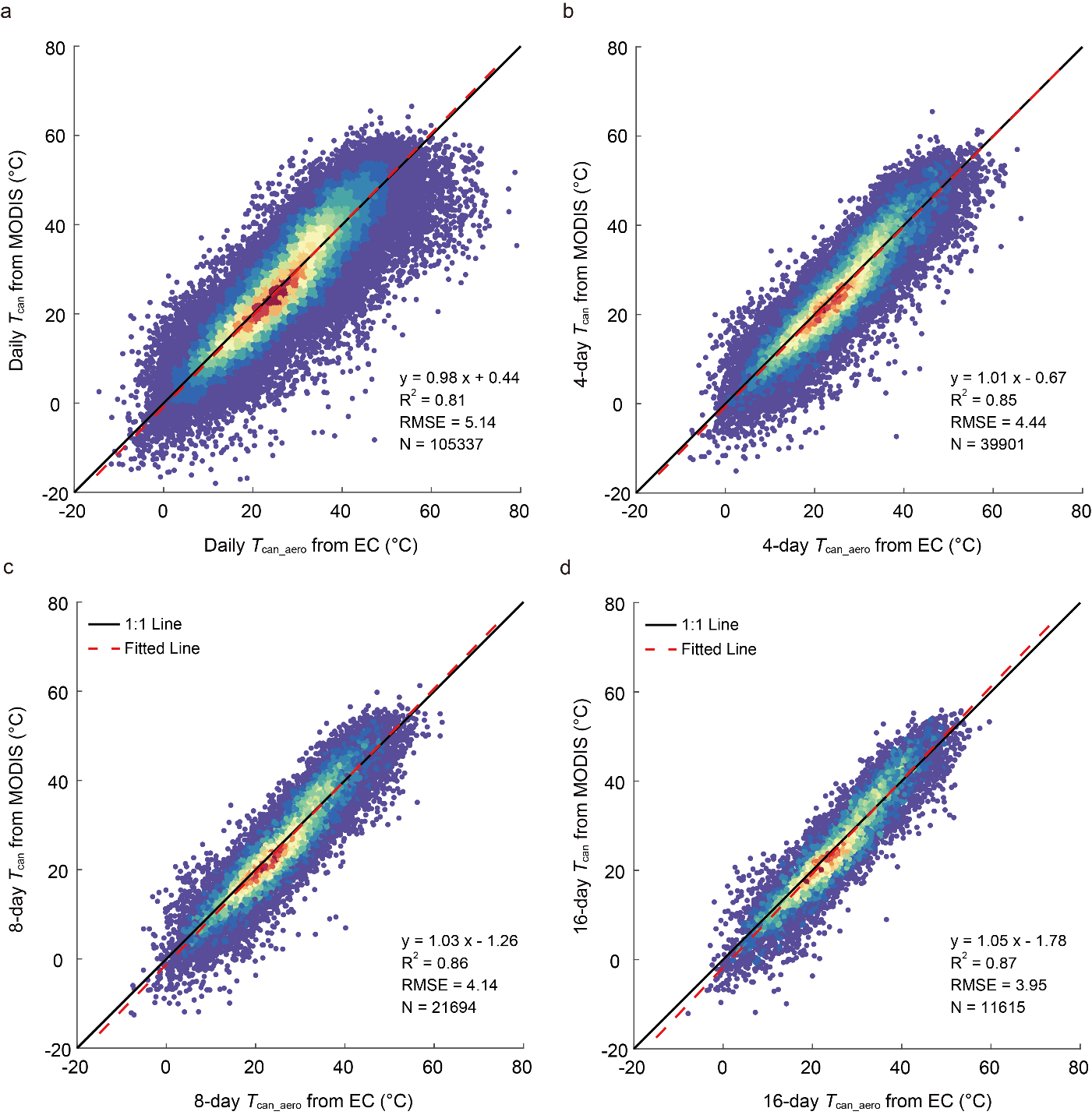
**

**Figure S24. The comparison between satellite-derived and eddy covariance (EC) canopy temperature across multiple temporal scales. a-d,** density scatterplots comparing satellite-derived *T*_can_ (“*y*”-axis) and EC-based *T*_can_aero_ (“*x*”-axis) aggregated at the 1-day, 4-day, 8-day, 16-day temporal scales. Each point represents a paired observation during the growing season, pooled across all valid sites. The solid black line denotes the 1:1 relationship, while the red dashed line indicates the linear regression fit. Statistical metrics are reported within each panel. Warmer colors indicate higher point density.

**
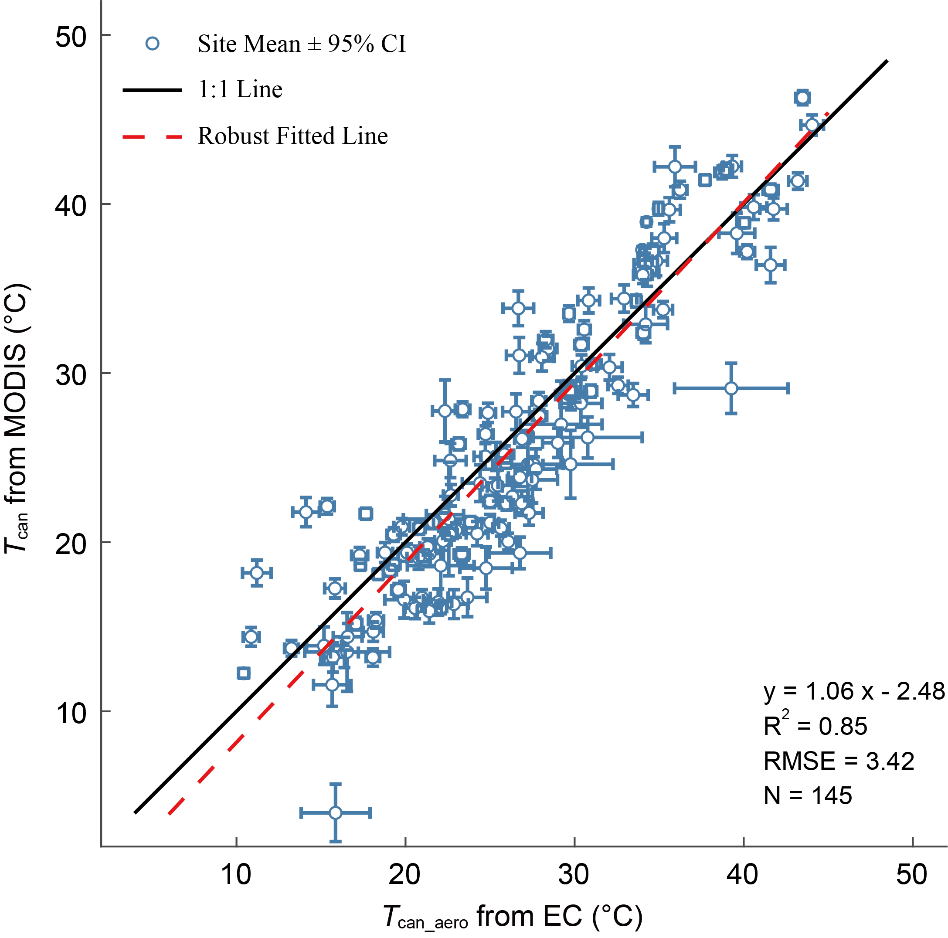
**

**Figure S25. The comparison between site-averaged canopy temperatures derived from MODIS satellite data and eddy covariance measurements.** Each open circle represents the mean canopy temperature for an individual site, averaged over the study period. Vertical and horizontal error bars denote the 95% confidence intervals for the MODIS-derived and EC-derived estimates, respectively. The solid black line indicates the 1:1 relationship, while the red dashed line represents the robust linear regression fit. Inset statistics provide the linear equation, coefficient of determination (R^2^), root mean square error (RMSE), and the number of sites (N).


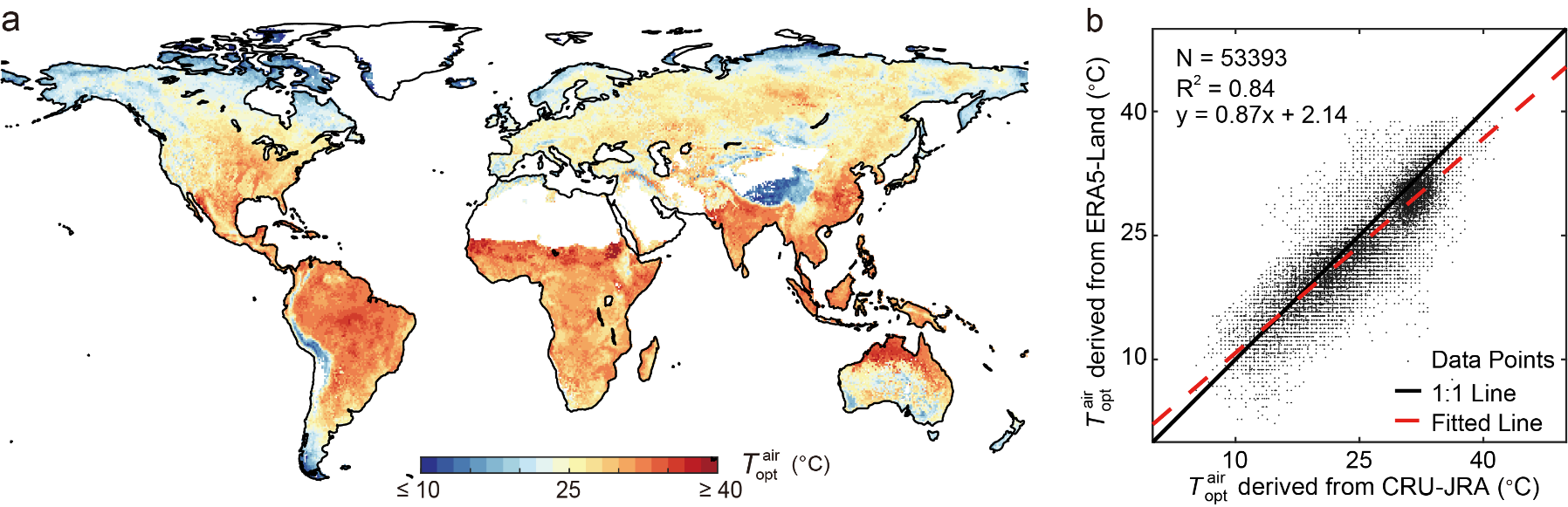


**Figure S26. Evaluation of ecosystem-scale *T*air opt for vegetation productivity using the CRUJRA reanalysis air temperature. a,** Spatial distribution of ecosystem-scale optimal air temperature (*T*air op**t**) estimated using CRU-JRA daily maximum air temperature for the period 2003–2023. **b,** desity scatterplots comparing CRU-JRA derived *T*air opt (“*x*”-axis) with EAR5-Land based *T*air opt (“*y*”-axis). The solid black line represents the 1:1 relationship, and the red dashed line indicates the linear regression fit.


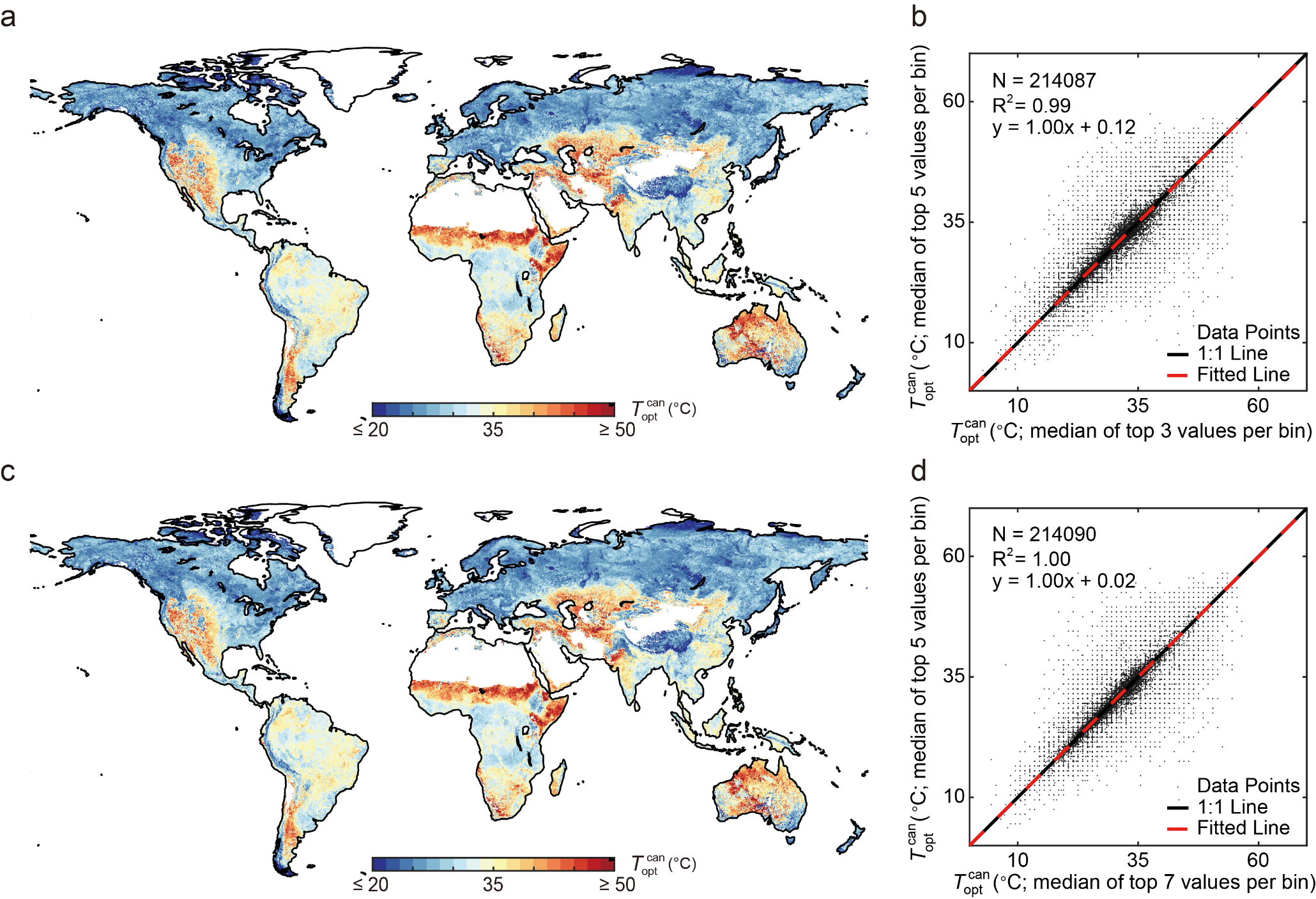


**Figure S27. Sensitivity of ecosystem-scale *T*can opt estimates to the selection of the upper-envelope threshold for productivity. a,** Spatial distribution of *T*can opt calculated using the median of the top three productivity values within each temperature bin. **b,** comparison of *T*can opt derived using the top three threshold (“*x*”-axis) against the baseline method (median of the top five values, “*y*”-axis) used in the main analysis. The solid black line represents the 1:1 relationship, and the red dashed line presents the linear regression fit. **c, d,** similar to **a, b,** but using the median of the top seven productivity values within each temperature bin.


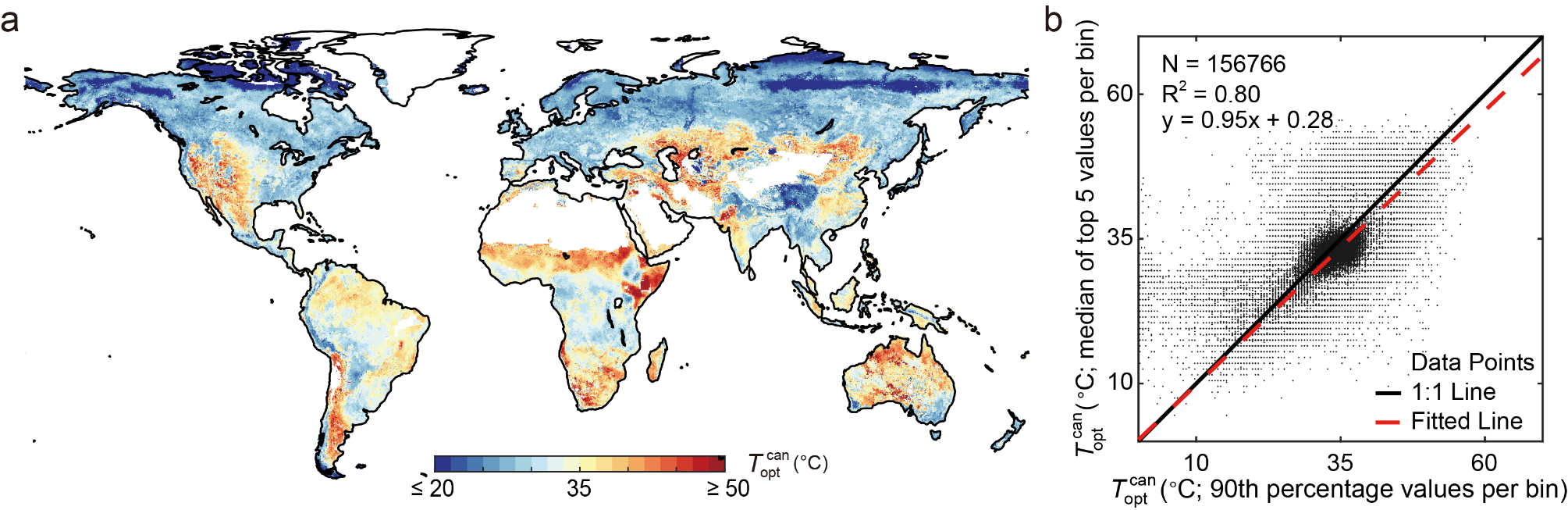


**Figure S28. Comparison of ecosystem-scale *T*can opt derived using the upper-envelope estimator versus the 90^th^ percentile estimator. a,** Spatial distribution of *T*can opt calculated using the 90^th^ percentile values within each temperature bin. **b,** comparison of *T*can opt derived using the 90^th^ percentile method (“*x*”-axis) against the baseline method (median of the top five values, “*y*”-axis) used in the main analysis. The solid black line represents the 1:1 relationship, and the red dashed line presents the linear regression fit.


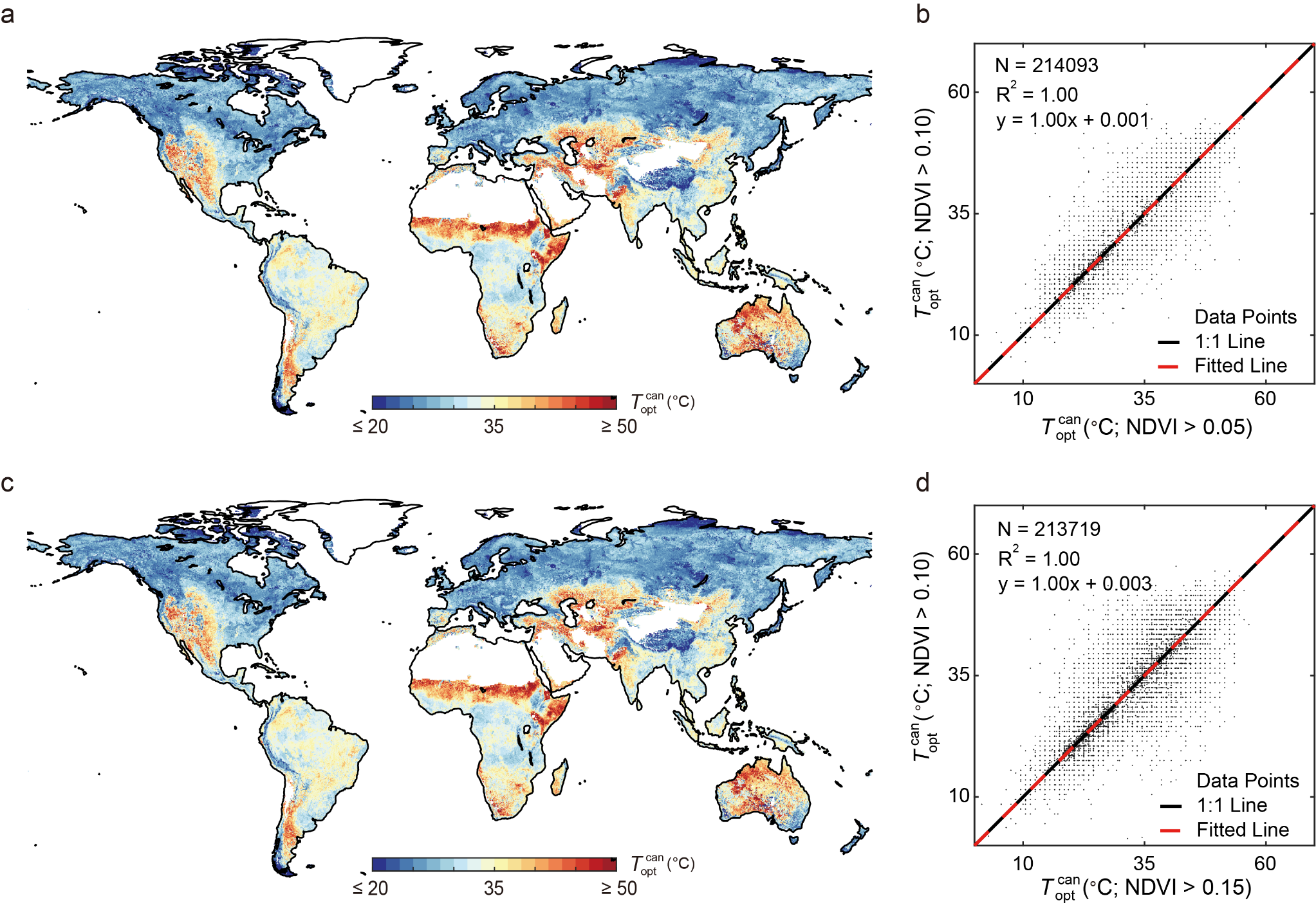


**Figure S29.** **Sensitivity of ecosystem-scale *T*can opt estimates to the selection of the annual mean NDVI thresholds.** **a,** Spatial distribution of *T*can opt calculated using an annual mean NDVI threshold of > 0.05. **b,** comparison of *T*can opt derived using the relaxed threshold (NDVI>0.05, “*x*”-axis) against the baseline threshold used in the main analysis (NDVI>0.10, “*y*”-axis). The solid black line represents the 1:1 relationship, and the red dashed line is the linear regression fit. **c, d,** similar to **a, b,** but using a more conservative vegetation threshold of NDVI > 0.15.


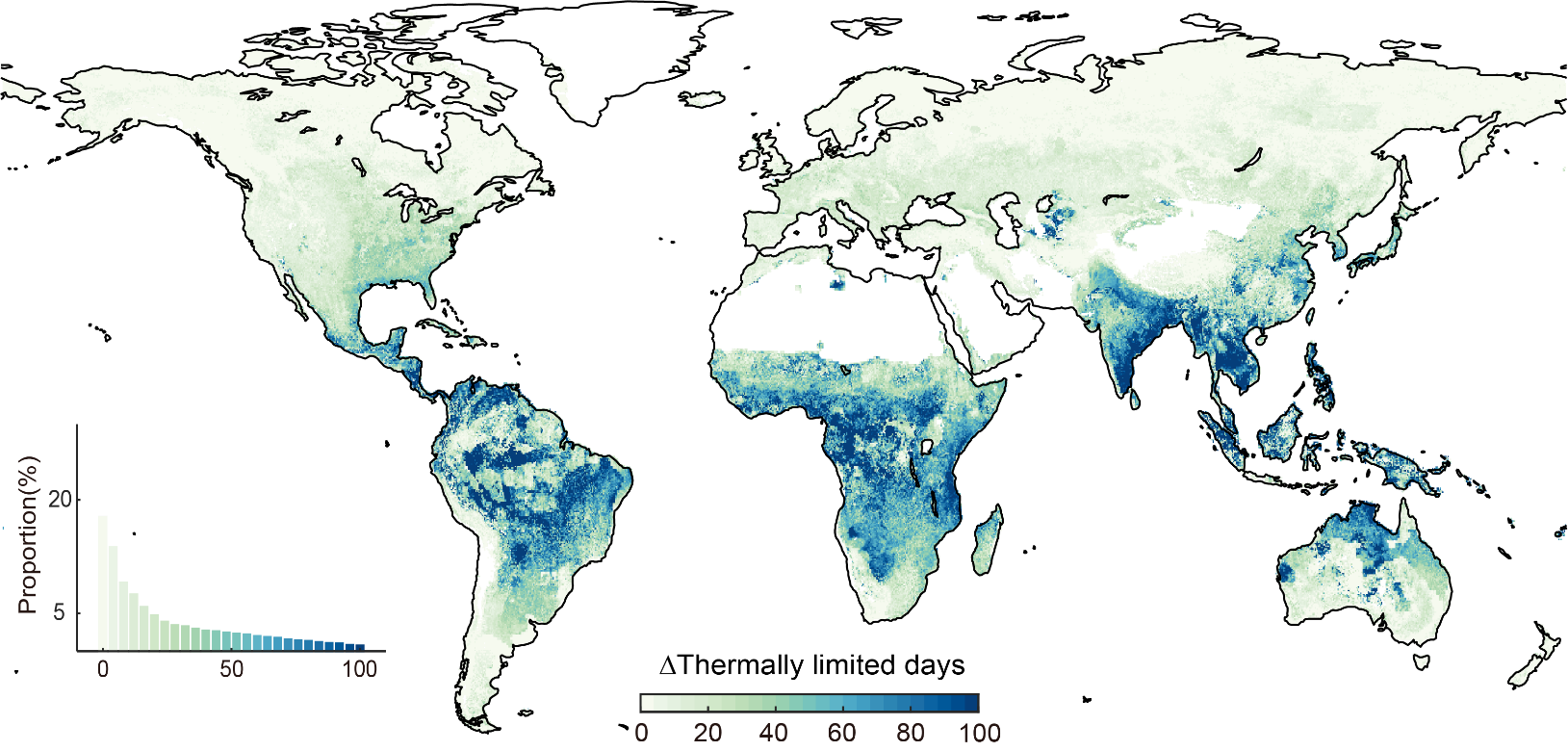


**Figure S30. Spatial distribution of the difference in the number of thermally limited days (ΔThermally limited days) between the gap-filled and raw (unfilled) canopy temperature datasets.** Positive values indicate areas where gap-filling successfully recovered expected heat stress events that would otherwise have been missed due to cloud gaps or other quality issues. The inset histogram shows the frequency distribution of these differences, confirming that neglecting gap-filling leads to a systematic underestimation of heat-stress duration.


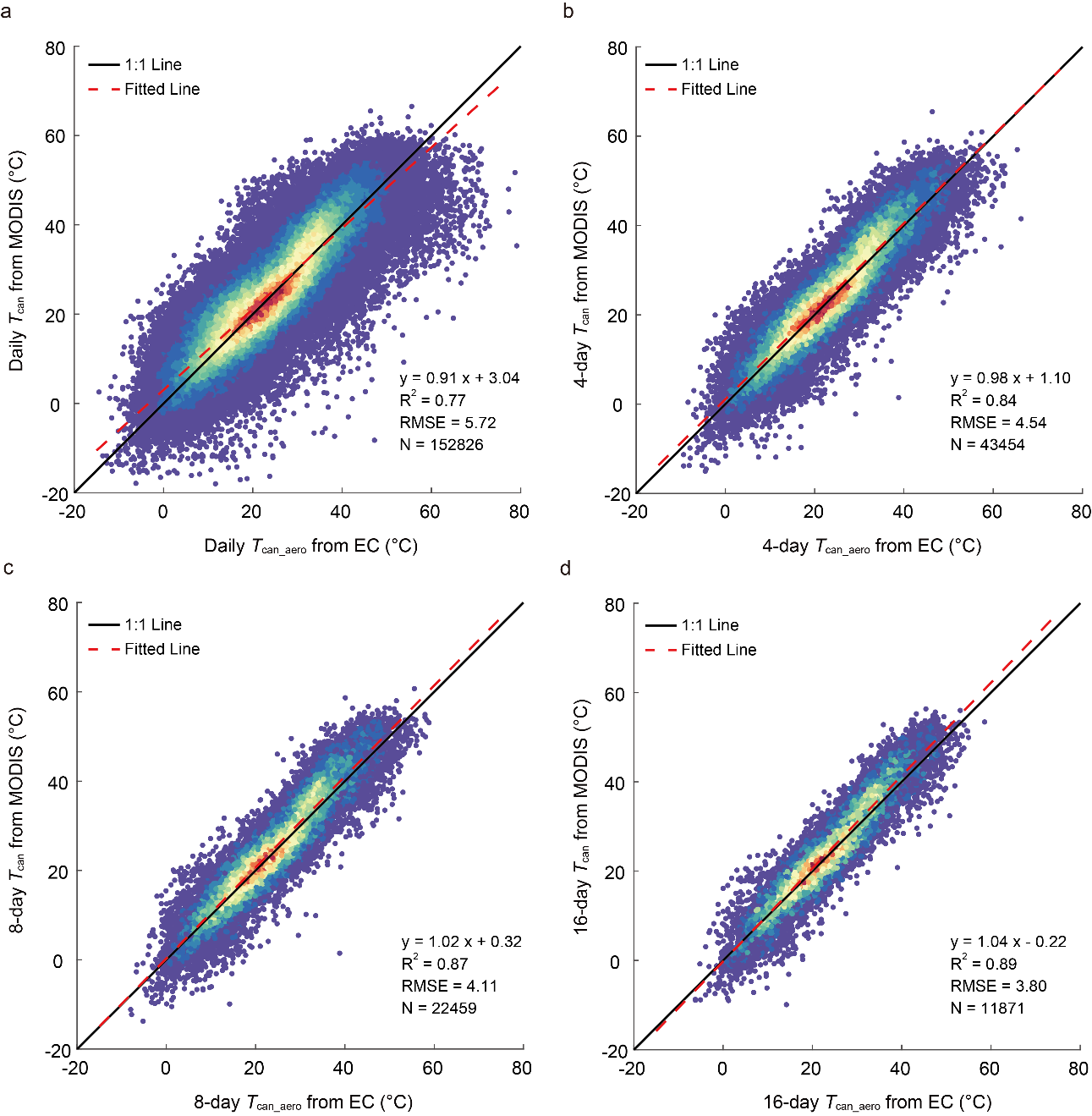


**Figure S31. The comparison between gap-filled satellite-derived and eddy covariance (EC)- canopy temperature across multiple temporal scales. a-d,** density scatterplots comparing satellite-derived *T*_can_ (“*y*”-axis) and EC-based *T*_can_aero_ (“*x*”-axis) aggregated at the 1-day, 4-day, 8-day, 16-day temporal scales. Each point represents a paired observation during the growing season pooled across all valid sites. The solid black line denotes the 1:1 relationship, while the red dashed line indicates the linear regression fit. Statistical metrics are reported within each panel. Warmer colors indicate higher point density.

**References**

1. Jiang M, Guo K, Wang J *et al.* Current status and prospects of rice canopy temperature research. *Food Energy Secur* 2023; **12**: e424.
2. Gauthey A, Bachofen C, Deluigi J *et al.* Absence of canopy temperature variation despite stomatal adjustment in *Pinus sylvestris* under multidecadal soil moisture manipulation. *New Phytol* 2023; **240**: 127–137.
3. Islam T, Hulley GC, Malakar NK *et al.* A Physics-Based Algorithm for the Simultaneous Retrieval of Land Surface Temperature and Emissivity From VIIRS Thermal Infrared Data. *IEEE Trans Geosci Remote Sens* 2017; **55**: 563–576.
4. Doughty CE, Keany JM, Weibe BC *et al.* Tropical forests are approaching critical temperature thresholds. *Nature* 2023; **621**: 105–111.
5. Hulley G, Freepartner R, Malakar N *et al*. Moderate Resolution Imaging Spectroradiometer (MODIS) Land Surface Temperature and Emissivity Product (MxD21) User Guide. [https://lpdaac.usgs.gov/documents/108/MOD21_User_Guide_V6.pdf](https://lpdaac.usgs.gov/documents/108/MOD21_User_Guide_V6.pdf.) (19 May 2026, date last accessed).
6. Huang M, Piao S, Ciais P *et al.* Air temperature optima of vegetation productivity across global biomes. *Nat Ecol Evol* 2019; **3**: 772–779.
7. Mildrexler DJ, Zhao M, Running SW. Satellite Finds Highest Land Skin Temperatures on Earth. *Bull Am Meteorol Soc* 2011; **92**: 855–860.
8. Didan K, Munoz AB, Solano R *et al*. MODIS Vegetation Index User’s Guide (MOD13 Series). <https://lpdaac.usgs.gov/documents/103/MOD13_User_Guide_V6.pdf> (19 May 2026, date last accessed).
9. Doughty CE, Goulden ML. Are tropical forests near a high temperature threshold? *J Geophys Res* 2008; **113**: 2007JG000632.
10. Guo Z, Still CJ, Lee CKF *et al*. Does plant ecosystem thermoregulation occur? An extratropical assessment at different spatial and temporal scales. *New Phytol* 2023; **238**: 1004–1018.
11. Pastorello G, Trotta C, Canfora E *et al*. The FLUXNET2015 dataset and the ONEFlux processing pipeline for eddy covariance data. *Sci Data* 2020; **7**: 225.
12. Friedlingstein P, O'Sullivan M, Jones MW *et al*. Global Carbon Budget 2020. *Earth Syst Sci Data* 2020; **12**: 3269–3340.
13. Qiu J, Zhang Y, Cai M *et al*. Large contribution of antecedent climate to ecosystem productivity anomalies during extreme events. *Nat Geosci* 2026; **19**: 25–32.
14. Running SW, Zhao M. Daily GPP and annual NPP (MOD17A2H/A3H) and year-end gap- filled (MOD17A2HGF/A3HGF) products NASA earth observing system MODIS land algorithm (for collection 6.1). <https://lpdaac.usgs.gov/documents/972/MOD17_User_Guide_V61.pdf> (19 May 2026, date last accessed).
15. Zhu Z, Piao S, Myneni Rb *et al*. Greening of the Earth and its drivers. *Nat Clim Change* 2016; **6**: 701–795.
16. Badgley G, Field CB, Berry JA. Canopy near-infrared reflectance and terrestrial photosynthesis. *Sci Adv* 2017; **3**: e1602244.
17. Dechant B, Ryu Y, Badgley G *et al*. NIRVP: A robust structural proxy for sun-induced chlorophyll fluorescence and photosynthesis across scales. *Remote Sens Environ* 2022; **268**: 112763.
18. Zhang, Y, Joiner J, Alemohammad SH *et al*. A global spatially contiguous solar-induced fluorescence (CSIF) dataset using neural networks. *Biogeosciences* 2018; **15**: 5779–5800.
19. Baldocchi DD, Ryu Y, Dechant B *et al*. Outgoing near‐infrared radiation from vegetation scales with canopy photosynthesis across a spectrum of function, structure, physiological capacity, and weather. *J Geophys Res Biogeo* 2020; **125**: e2019JG005534.
20. Wu G, Guan K, Jiang C *et al.* Radiance-based NIR_v_ as a proxy for GPP of corn and soybean. *Environ Res Lett* 2020; **15**: 034009.
21. Zhang X, Liang S, Zhou G *et al*. Generating Global Land Surface Satellite incident shortwave radiation and photosynthetically active radiation products from multiple satellite data. *Remote Sens Environ* 2014; **152**: 318-332.
22. Muñoz Sabater, J. et al. ERA5-land post-processed daily-statistics from 1950 to present. <https://cds.climate.copernicus.eu/datasets/derived-era5-land-daily-statistics?tab=overview> (19 May 2026, date last accessed).
23. NOAA National Centers for Environmental Information*.* Global Precipitation Climatology Project (GPCP) Climate Data Record (CDR), Version 1.3 (Daily). <https://www.ncei.noaa.gov/access/metadata/landing-page/bin/iso?id=gov.noaa.ncdc:C00999#Documentation> (19 May 2026, date last accessed).
24. Harris I, Osborn TJ, Jones P *et al*. Version 4 of the CRU TS monthly high-resolution gridded multivariate climate dataset. *Sci Data* 2020; **7**: 109.
25. Kobayashi S, Ota Y, Harada Y *et al*. The JRA-55 reanalysis: general specifications and basic characteristics. *J Meteor Soc Japan* 2015; **93**: 5–48.
26. Wang Y, Sarmah S, Singha M *et al*. Increasing optimum temperature of vegetation activity over the past four decades. *Earth Future* 2024; **12**: e2024EF004489.
